# Lattice Dynamics of van der Waals Layered Rhodium Chalcochlorides: Janus RhSeCl and 1.5-Dimensional RhTeCl

**DOI:** 10.1021/acs.inorgchem.6c02052

**Published:** 2026-08-10

**Authors:** Sandra Schiemenz, Domenic Nowak, Samuel Froeschke, Marco Naumann, Marco Rosenkranz, Bernd Büchner, Martin Knupfer, Nico Gräßler, Silke Hampel, Stanislav M. Avdoshenko, Alexey A. Popov

**Affiliations:** 28394 Leibniz Institute for Solid State and Materials Research Dresden (IFW Dresden), Dresden 01069, Germany

## Abstract

van der Waals layered rhodium chalcochlorides demonstrate a unique interplay between the structure of the layers and the size of the anions. While RhSeCl has a 2D Janus structure with Se and Cl on opposite sides of the layers, RhTeCl forms a non-Janus structure with alternating stripes of Cl and Te. Here, we study the lattice dynamics of these semiconductors by a combination of far-infrared and Raman spectroscopy and DFT phonon calculations. Polarization-dependent measurements enabled the complete assignment of all Raman-active modes of both compounds. The resonance effects in Raman scattering were addressed by using multiple excitation wavelengths and a tunable dye laser between 457 and 785 nm. Raman excitation profiles appeared to be more sensitive to optical excitations than direct measurements of excitation spectra by diffuse reflectance spectroscopy. Finally, variable-temperature Raman measurements between 10 and 300 K revealed that the optical phonons of RhSeCl are weakly susceptible to temperature variations, whereas the temperature dependence of RhTeCl is more pronounced.

## Graphical abstract

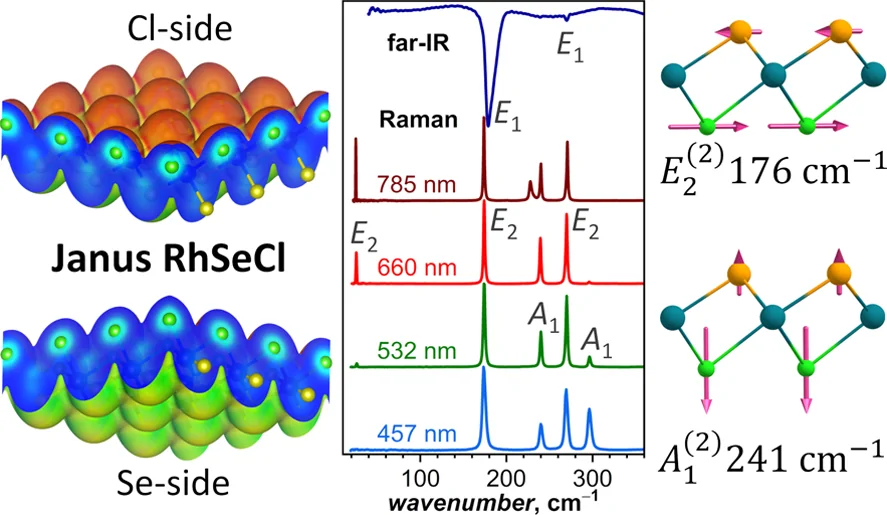

## Introduction

Functional inorganic solid-state materials play a crucial role in tackling modern technological challenges, ranging from energy storage^1^ to advanced catalysis.^2^ While historical efforts to optimize material properties have predominantly focused on the variation of cations, the targeted manipulation of anions has emerged as a powerful frontier in materials science.^3^ The deliberate combination of different anions yields heteroanionic (or mixed-anion) compounds, which often exhibit distorted coordination polyhedra that enable the precise tuning of electronic, magnetic, and optical properties.^4,5^

Within this domain, two-dimensional (2D) Janus materials represent a particularly distinct and highly functional subclass of van der Waals structures.^6–11^ Deriving their name from the two-faced Roman god, these heteroanionic 2D materials feature different functionalizations, such as distinct atomic anions, segregated on opposing sides of a single atomic layer. This specific structural order fundamentally breaks the material’s mirror symmetry, which, in turn, induces a vertical intrinsic polarization field across the unit cell. This internal field is a direct consequence of the unbalanced charge distribution driven by the significant electronegativity difference between the top and bottom layers of atoms. Theoretical models and emerging empirical data suggest that this broken symmetry unlocks novel quantum mechanical and physical phenomena, including Rashba splitting and vertical piezoelectricity.^12–15^

Among the transition-metal chalcogenides, the 2D materials RhSeCl^16–18^ and its recently fully described structural non-Janus congener, RhTeCl,^19,20^ serve as compelling model systems for understanding these heteroanionic effects (Figure 1). Both compounds exhibit diamagnetic behavior and feature rhodium in a stable Rh­(III) oxidation state, yet their electronic structures are highly dependent on their specific anion orders. Spectroscopic investigations demonstrated that RhSeCl, a noncentrosymmetric Janus material, is a semiconductor with an indirect band gap of approximately 1.4 eV and exhibits pronounced second harmonic generation.^16,17,21,22^ By substituting selenium with tellurium, a less electronegative anion, the structure becomes non-Janus, while the band gap is significantly reduced to 0.8 eV in RhTeCl.^20^ This supports the fundamental principle that anions with lower electronegativities increase the covalent character and decrease the band gap. While the electronic structures of RhSeCl and RhTeCl have already been addressed in recent works, their vibrational properties remain largely unknown. Here, we focus on the lattice dynamics of RhSeCl and RhTeCl by IR and variable-excitation and variable-temperature Raman spectroscopy, supported by DFT calculations.

**Figure 1:**
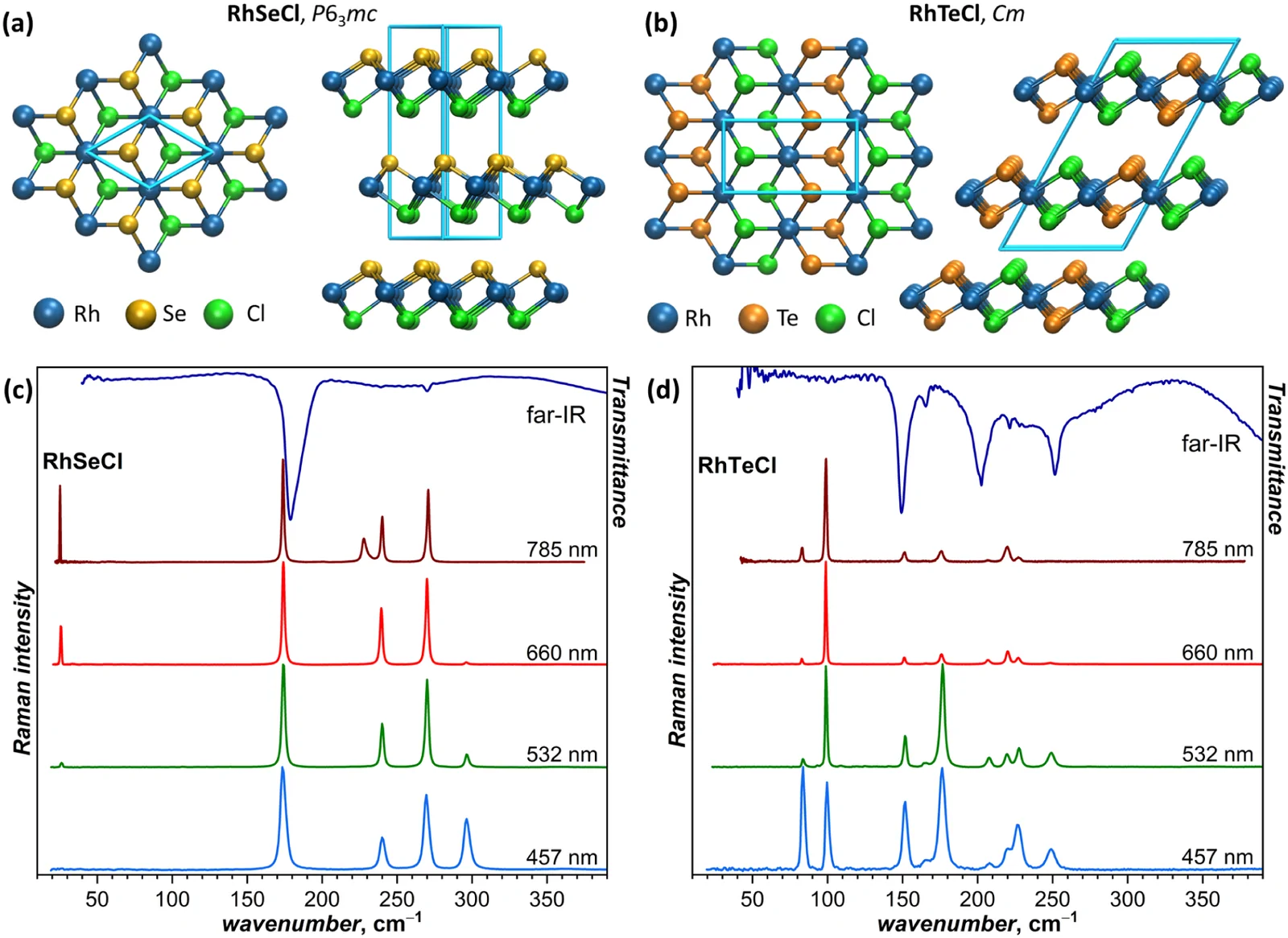
(a, b) Structures of isolated layers and packing of the layers in the crystals of (a) RhSeCl and (b) RhTeCl; unit cells are visualized in light cyan. (c, d) Far-IR and Raman spectra of (c) RhSeCl and (d) RhTeCl. IR spectra were measured for powder samples, while Raman spectra were obtained for single crystals and excited by four different lasers. All spectra were measured at room temperature.

## Experimental and Computational Details

### Synthesis and Crystal Growth of RhSeCl and RhTeCl

The first crystal growth of RhSeCl, reported by our group in 2023, produced crystals of relatively small size.^16^ To increase the size of the crystals, we modified the synthesis procedure. The growth of RhSeCl single crystals in this work was conducted using the oscillating temperature profile chemical vapor transport (OTP-CVT) method.^23^ This approach optimizes crystal growth by dynamically varying gas-phase saturation, where rapid heating phases increase the solubility of the starting materials, and subsequent slow cooling phases promote high-quality single-crystal formation. For the preparation, a stoichiometric mixture consisting of rhodium, rhodium­(III) chloride, and selenium was utilized in a 2:1:3 molar ratio. To ensure the purity of the deposition zone, an initial cleaning transport was performed for 12 h using a reversed thermal gradient with temperatures of *T*_1_ = 1000 °C and *T*_2_ = 900 °C to remove any contaminants from the sink area. Following this cleaning step, both the source and sink zones were adjusted to their starting temperatures of *T*_1_ = 888 °C and *T*_2_ = 988 °C. The primary synthesis process consisted of 27 periodic cycles, with each complete heating and cooling cycle lasting 8 h. During the heating phase, both furnace zones were ramped at a rate of 12 K/h until reaching target temperatures of *T*_1_ = 900 °C and *T*_2_ = 1000 °C. This was immediately followed by a cooling phase at a rate of −4 K/h until the temperatures reached *T*_1_ = 876 °C and *T*_2_ = 976 °C (Figure S1). After the completion of all 27 cycles, the ampule was allowed to cool naturally to room temperature. This methodology successfully produced metallic, shining RhSeCl crystals in the millimeter size range within the sink region of the ampule (Figure S2). The recovered crystals were cleaned by rinsing the ampule with isopropanol and placing it in an ultrasonic bath for 2 min to effectively detach the crystals from the quartz glass walls.

For the synthesis of RhTeCl, the method of Köhler and Urland^19^ was adjusted by using tellurium tetrachloride as a source of chlorine. Powder X-ray diffraction and spectroscopic characterization of the thus prepared RhTeCl were reported recently in ref 20.

### Spectroscopic Characterization

Raman spectra were studied using a T64000 spectrometer (Horiba Jobin Yvon) with 1800 gr/mm gratings, equipped with a microscope, a Symphony CCD detector with liquid nitrogen cooling, an LT3-OM cryostat (Advanced Research Systems), and solid-state excitation lasers with wavelengths of 457 nm (Cobolt Twist model by Hübner Photonics), 532 and 660 nm (Torus models by Laser Quantum Novanta Photonics), and 785 nm (BrixX diode laser by Omicron-Laserage Laserprodukte). Besides, a dye laser, Matisse 2 (by Sirah Lasertechnik), charged with Rhodamine 6G or 2-methyl-6-(4-dimethylaminostyryl)-4H-pyran (DCM) and pumped by a 532 nm laser (Millennia by Spectra-Physics), was used to tune the excitation wavelength between 570 and 720 nm. Vibrational frequencies were calibrated against the Si peak at 25 °C, which is located at 520.6 cm^–1^ following the analysis performed by Itoh.^24^ In the measurements denoted as nonpolarized, the excitation laser beam was depolarized with a polarization scrambler. In linearly polarized measurements, the polarization plane of the laser was rotated with a polarization rotator, while the analyzer placed before the detector was fixed either vertically or horizontally.

Far-IR spectra were recorded at room temperature in transmission mode using a Vertex 80v spectrometer (Bruker). Powders of RhSeCl and RhTeCl were rubbed onto a polyethylene film, and the spectra were recorded with a SiC Globar light source and a DLaTGS detector with a polyethylene window. Diffuse reflectance measurements of powder samples pressed into BaSO_4_ pellets were performed by using a Shimadzu UV-3101PC spectrometer.

Photoluminescence measurements were performed in backscattering geometry using a modified fluorescence microscope (Olympus BX series), laser excitation at 488 nm (Omicron PhoxX diode laser), a Kymera 328i spectrograph (Andor), and an iDus 1.7 μm InGaAs camera (Andor), with intensity-calibrated against the blackbody radiation of a tunable halogen lamp. The temperature of the sample was controlled using a Janis ST-500 microscopy cryostat.

### Computational Studies

DFT calculations of vibrational spectra were performed with VASP 5.4.4,^25–27^ using the PBE^28^ functional with and without dispersion correction D3,^29^ corresponding pseudopotentials, 520 eV energy cutoffs for the projector-augmented wave (PAW) scheme, and a 4 × 2 × 4 Γ-centered k-grid. Hessian matrices were calculated using density-functional perturbation theory for a 2 × 2 × 2 supercell; phonons were then calculated and analyzed using in-house Python scripts and Phonopy libraries.^30^ Atomic charges were computed using the code Bader.^31^ Molecular structures, isosurfaces, and vibrational displacements were visualized with VMD,^32^ Vesta,^33^ and Chemcraft^34^ codes.

## Results and Discussion

### Vibrational Spectra of RhSeCl

In RhSeCl, the first coordination sphere of Rh includes three Se and three Cl atoms in a trigonal-antiprism arrangement (Figure 1a). These distorted octahedra are connected by edges to form a layer, in which Se atoms appear on one side and Cl atoms on the other side of the Rh sublayer. Thus, RhSeCl layers have a Janus structure. The layers are stacked in an AB manner so that the Cl side of one layer interfaces with the Se side of the next layer, and the resulting crystal structure is described by the hexagonal *P*6_3_ *mc* (186) space group (CCDC 2231776).^16^ The 6_3_-screw symmetry axis is perpendicular to the layers.

The isolated RhSeCl layer is described by the *C*_3*v*_ point symmetry with 3*A*_1_ + 3*E* vibrational modes, of which *A*_1_ + *E* would have zero frequency at the Γ-point. AB-type stacking of these layers in the crystal gives the *C*_6*v*_ factor group and doubles the number of modes, with each layer mode giving two modes in the crystal: *A*_1_ → *A*_1_ + *B*_1_, *E* → *E*_1_ + *E*_2_. Vibrations of the RhSeCl crystal thus span the following irreducible representations of the *C*_6*v*_ group:

$$\Gamma_{\mathrm{vib}}\left(C_{6v}\right)=3A_{1}+3B_{1}+3E_{1}+3E_{2}$$

*A*_1_, *E*_1_, and *E*_2_ modes are Raman-active, but *E*_1_ will remain silent in the backscattering geometry with excitation laser shining normal to the layers, as used in this work. *A*_1_ and *E*_1_ modes are also active in IR absorption, while *B*_1_ is silent in both IR and Raman. Acoustic modes of the layer produce not only acoustic modes of the crystal (*E*_1_ + *A*_1_), but also the interlayer shear mode (*E*_2_) and the layer breathing mode (*B*_1_). Overall, 2*A*_1_ + 3*E*_2_ modes are expected in Raman and 2*A*_1_ + 2*E*_1_ in IR spectra of RhSeCl.

Figure 1c compares the far-IR spectra of the powdered RhSeCl and the Raman spectra of the crystal. To probe the presence of resonance effects, four laser wavelengths—457 nm (2.71 eV), 532 nm (2.33 eV), 660 nm (1.88 eV), and 785 nm (1.58 eV)—were used. The IR spectrum has only two visible absorption bands: one strong at 179 cm^–1^ and one much weaker at 270 cm^–1^. The Raman spectra exhibit altogether 6 lines, one more than the number of expected fundamental modes, and the spectral appearance varies considerably with the excitation energy. Whereas the peaks at 176, 241, and 272 cm^–1^ retain similar relative intensities in all three spectra, the low-frequency line at 26 cm^–1^ transforms from the second strongest peak in the spectrum excited at 785 nm to a barely visible weak feature when excited at 532 nm. The peak at 229 cm^–1^ is visible only with the near-IR excitation, while the feature at 272 cm^–1^ is absent at 785 nm but appears at 660 nm and acquires higher intensity at 532 and 457 nm. The variation of the spectra with excitation energy points to resonance effects, which will be discussed further below. The frequencies of the IR bands and two of the Raman peaks are very close, which is caused by their origin from the same vibrations of a single layer.

To aid in the symmetry assignment of the vibrational modes, we performed linearly polarized Raman measurements (Figure 2a). Both *A*_1_ and *E*_2_ modes are active when the polarization plane of the laser is parallel to the analyzer before the detector (denoted as *XX* or *YY*; light propagates along *Z*), while only *E*_2_ modes remain active in the cross-polarized configuration. The virtual disappearance of the peaks at 241 and 299 cm^–1^ in *XY* and *YX* configurations allowed their straightforward assignment as $A_{1}^{\left(2\right)}$ and $A_{1}^{\left(3\right)}$ modes, respectively ( $A_{1}^{\left(1\right)}$ is the acoustic phonon in this numbering system). The assignment was further substantiated by the rotation of the laser polarization plane in 10° steps (Figure 3). The angular variation of the intensity for these modes and the nearly constant intensity of the peaks at 176 and 272 cm^–1^ confirmed the attribution of the former as *A*_1_ modes and the latter as *E*_2_ modes. Interestingly, as-grown mm-sized crystals of RhSeCl occasionally showed some residual intensity of *A*_1_ modes in the cross-polarized configuration, suggesting the intergrowth of single-crystalline grains with different in-plane orientations. The polarization of *A*_1_ modes was improved when the crystals were thinned down with the Scotch-tape technique to a few nm, which also reduced their lateral dimensions to some tens of μm (Figure S3). These smaller and thinner crystals demonstrated not only cleaner polarization but also considerably increased Raman intensity. Note that Raman spectra of monolayer RhSeCl, recently reported in ref 18, retained peak positions and intensities similar to those in the bulk crystals.

**Figure 2:**
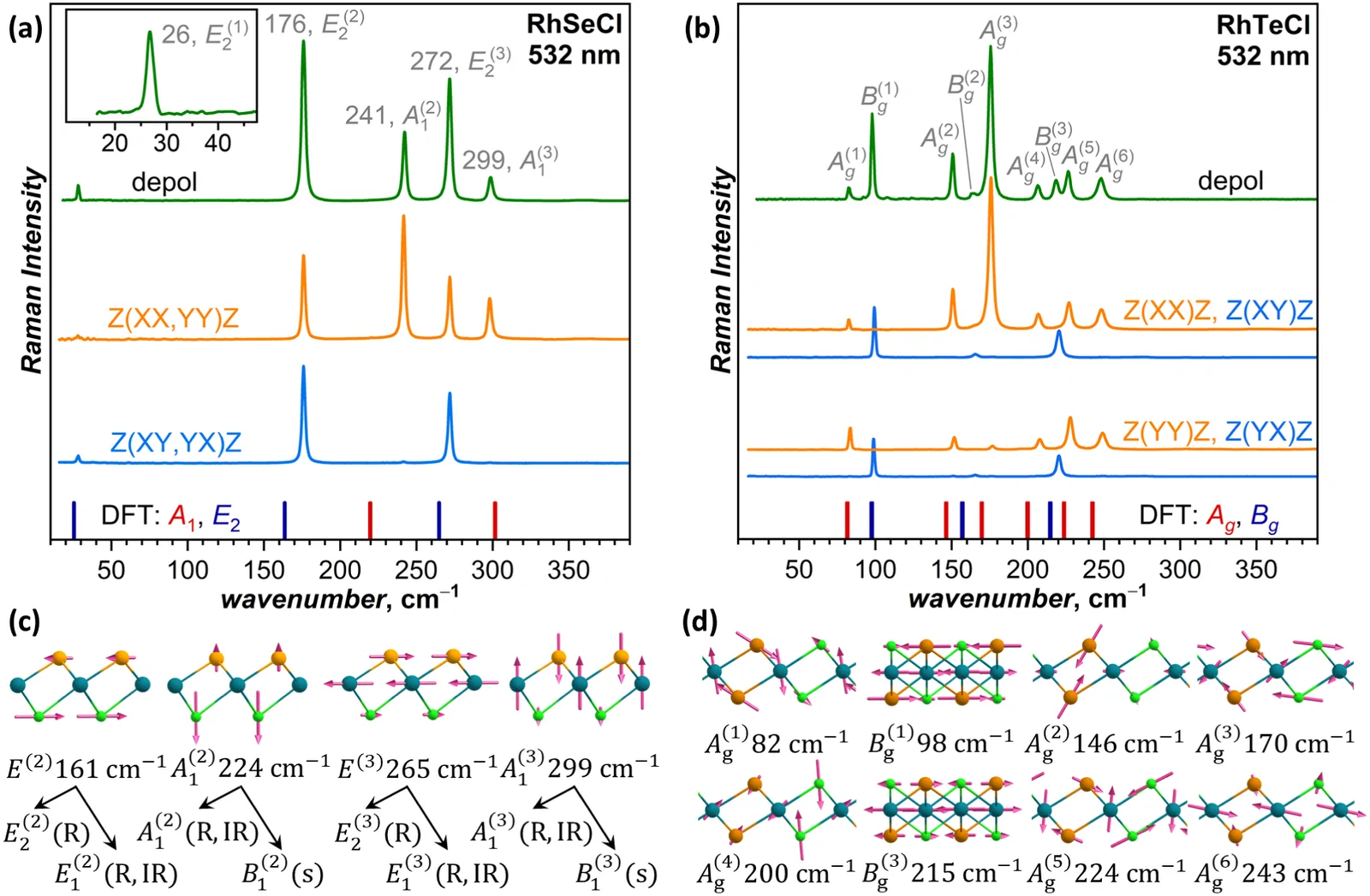
(a) Nonpolarized and linearly polarized Raman spectra of RhSeCl (room temperature, excitation at 532 nm) and DFT-calculated frequencies of Raman-active modes in Γ-point. (b) Nonpolarized and linearly polarized Raman spectra of RhTeCl (excitation at 532 nm) and DFT-calculated frequencies of Raman-active modes in Γ-point. (c) DFT-calculated vibrational frequencies and displacements of the RhSeCl layer (*C*_3*v*_) and relations between normal modes of a single layer and a crystal (*C*_6*v*_); letters in parentheses denote activity in the spectra: R – Raman, IR – infrared, s – silent. (d) DFT-calculated vibrational frequencies and displacements of Raman-active modes in the RhTeCl crystal (irreducible representations are given for the *C*_2*h*_ symmetry).

**Figure 3:**
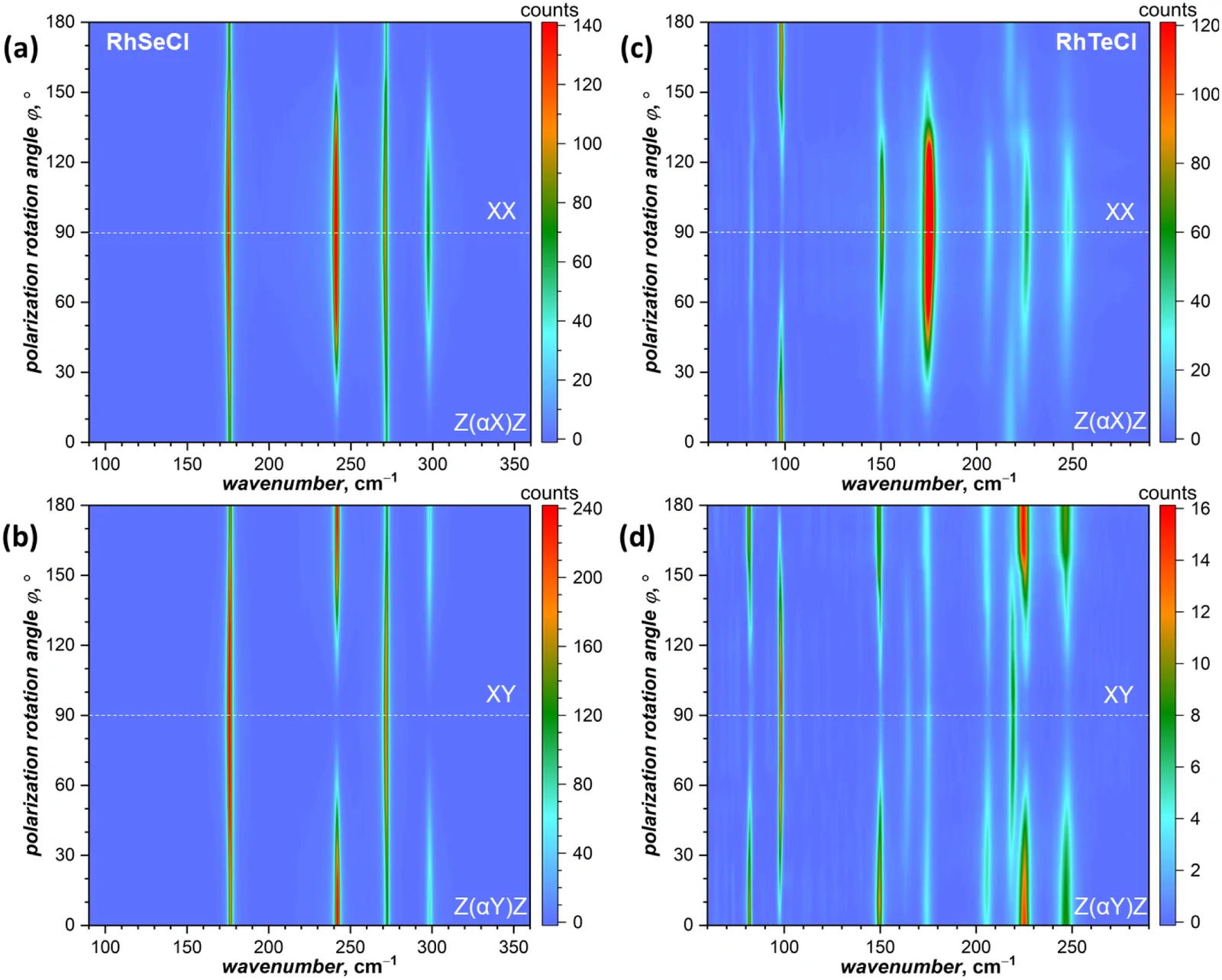
Raman intensity profiles for the rotation of the laser polarization plane in the spectra of (a, b) RhSeCl and (c, d) RhTeCl. The polarization plane of the laser was rotated in 10° steps, and the analyzer on the detector side was set either vertically (a, c) or horizontally (b, d). Room temperature, excitation at 532 nm.

We also performed DFT calculations to guide the assignment of vibrational modes and identify expected frequencies of silent modes. To evaluate the role of dispersion interaction, computations were performed with PBE and PBE-D3 functionals (see Table S1 for the full set of frequencies). If the cell parameters are fixed to their experimental values and only internal coordinates are optimized, the difference between PBE and PBE-D3 frequencies does not exceed 3.1 cm^–1^. When cell parameters were allowed to relax as well, the cell parameter *c* (equal to twice the interlayer distance) is predicted by PBE and PBE-D3 to be 11.612 and 11.417 Å, respectively, which can be compared to the experimental value of 11.579 Å determined at 293 K.^16^ When compared to WS_2_ ^35^ or CrCl_3_,^36^ for which PBE overestimated the interlayer distance by ∼1 Å, whereas PBE-D3 was much closer to experimental values, the difference between PBE and PBE-D3 found here for RhSeCl is comparably small. Likewise, vibrational frequencies predicted with and without D3 correction are also very similar. Based on these findings, we can conclude that Coulomb interactions play the main role in the interlayer bonding of layered rhodium chalcochlorides.

PBE-computed vibrational frequencies closely agree with experimental values, confirming the identification of Raman peaks and allowing the assignment of both IR peaks to *E*_1_ modes (Table 1), whereas the IR intensity of *A*_1_ modes appears too low for their reliable detection. The Raman peak at 228 cm^–1^ cannot be assigned to a fundamental mode and is caused either by the activation of a non-Γ-point phonon or by a combinational mode. For the latter option, a sum of $E_{1}^{\left(2\right)}$ (exp. 179 cm^–1^) and $B_{1}^{\left(1\right)}$ (calc. 49 cm^–1^) gives the only reasonable match, both by energy and by symmetry (*E*_1_×*B*_1_ = *E*_2_).

**Table 1:** DFT-Computed Vibrational Frequencies (cm^–1^) of the Layer and Crystal RhSeCl and Their Comparison to Experimental Far-IR and Raman Spectroscopic Data^a^ ^b^

**DFT layer,** ***C***_**3** ***v***_		**DFT crystal,** ***C***_**6** ***v***_		**Exp. far-IR**		**exp. Raman** **532/785** **nm**		*χ* ^c^ **(cm** ^**–1**^ **/K)**
*E* ^(1)^	0.0	$E_{1}^{\left(1\right)}$	0.0					
		$E_{2}^{\left(1\right)}$	25.4			26	vw/s	
$A_{1}^{\left(1\right)}$	0.0	$A_{1}^{\left(1\right)}$	0.0					
		$B_{1}^{\left(1\right)}$	49.1					
*E* ^(2)^	161.0	$E_{2}^{\left(2\right)}$	163.6			176	vs/vs	–0.0062(4)
		$E_{1}^{\left(2\right)}$	164.6	179	vs			
		$E_{1}^{\left(2\right)}+B_{1}^{\left(1\right)}$				229	–/m	
$A_{1}^{\left(2\right)}$	223.9	$A_{1}^{\left(2\right)}$	219.7			241	m/m	–0.0048(4)
		$B_{1}^{\left(2\right)}$	226.1					
*E* ^(3)^	264.8	$E_{1}^{\left(3\right)}$	264.0	270	w			
		$E_{2}^{\left(3\right)}$	264.9			272	s/s	–0.0067(4)
$A_{1}^{\left(3\right)}$	299.0	$A_{1}^{\left(3\right)}$	301.7			299	w/–	–0.0054(4)
		$B_{1}^{\left(3\right)}$	304.4					

a DFT refers to the PBE functional and computations with unit cell parameters fixed to their experimental values.

b Relative intensity scale is as follows: vs – very strong, s – strong, m – medium, w – weak, vw – very weak.

c χ stands for the first-order temperature (FOT) coefficient, determined as the slope of a linear fit.

Atomic vibrational displacements of the isolated RhSeCl layer (Figure 2b) are normal to the layer for *A*_1_ modes and in-plane for *E* modes. In the crystal, *A*_1_/*B*_1_ modes inherit perpendicular displacements from the *A*_1_ modes of the single layer, differing only in the vibrational phase between the two layers of the unit cell. The absence of *A*_1_ modes in the IR spectrum may, in fact, be a texture effect, as the crystallites in the powder are likely to be micro/nanosheets, which is not favorable for IR excitations of *A*_1_ vibrations, requiring an electric field component perpendicular to the layer. *E*_1_/*E*_2_ modes have in-plane displacements with opposite phases for *E*_1_ and *E*_2_. Somewhat counterintuitively, the lower-frequency vibrations at 176, 179, and 241 cm^–1^ are mainly displacements of Cl atoms, despite their much lower mass, whereas those above 250 cm^–1^ are oscillations of Rh and Se atoms moving counter to each other.

### Vibrational Spectra of RhTeCl

Similar to RhSeCl, layers of RhTeCl are also built from edge-sharing trigonal antiprisms formed by three Te and three Cl atoms around Rh. However, the orientation of coordination antiprisms in RhSeCl and RhTeCl is different. While in RhSeCl the 3-fold axes of antiprisms are oriented perpendicular to the layer, in RhTeCl the axes are only slightly tilted from the layer plane (Figure 1b). The 3-fold symmetry is therefore lost, and the RhTeCl crystal, with AB stacking of the layers, is described by the monoclinic space group *Cm* (8) (405714-ICSD).^19^ Since Cl and Te atoms are located both above and below Rh sublayers, RhTeCl has no Janus character. Instead, Cl and Te atoms form stripes along the *c*-direction of the crystal, which alternate along *b*.

The factor group symmetry of the RhTeCl crystal with the space group *Cm* (8) is *C*_*s*_, giving 12*A*’ + 6*A*” vibrational modes, all IR and Raman active (excluding 3 acoustic phonons). However, the *Cm* (8) structure of RhTeCl in ref 19 is only slightly distorted from the higher-symmetry structure with the *C*2/*m* (12) space group and *C*_2*h*_ factor group, the difference being in some Rh–Te and Rh–Cl bond lengths (Figure S4). Our DFT optimization, which used experimental atomic coordinates as the initial structural guess, resulted in the *C*2/*m* (12) structure (Figure S4). Similar to RhSeCl, the use of either PBE or PBE-D3 functional with dispersion correction resulted in similar geometries (Figure S4, Table S2), and we will further use results of PBE calculations with the unit cell parameters fixed to their experimental values from ref 19. Under the *C*_2*h*_ point symmetry, the vibrational representation of RhTeCl takes the form:

$$\Gamma_{\mathrm{vib}}\left(C_{2h}\right)=6A_{g}+3B_{g}+3A_{u}+6B_{u}$$

Here, *A_g_* and *B_g_* modes are Raman active (9 in total), *A_u_* +2*B_u_* are acoustic, and the remaining *A_u_* and *B_u_* modes are IR active (6 in total). Irreducible representations of *C*_2*h*_ and *C*_*s*_ groups are related as follows: *A_g_* ↔ *A*’, *A_u_* ↔ *A*”, *B_g_* ↔ *A*”, *B_u_* ↔ *A*’. As will be seen from the discussion below, Raman and some IR peaks correspond well to the Raman/IR activity expected for the *C*_2*h*_ symmetry, and we will keep the higher symmetry in the analysis. Note that the symmetry axis is aligned parallel to the layers, rather than perpendicular to them as in RhSeCl. An isolated layer of RhSeCl also has *C*_2*h*_ symmetry and the same number of formula units in the primitive cell as the crystal.

Far-IR and Raman spectra of RhTeCl are compared in Figure 1d. The spectra are richer than those of RhSeCl, as might be expected from the lower symmetry and the twice larger number of atoms in the unit cell. The IR spectra have 3 medium-strong bands at 149, 203, and 252 cm^–1^, and two weaker features at 165 and 221 cm^–1^. Raman spectra excited at 785 and 660 nm are dominated by the strong line at 99 cm^–1^, but the situation changes at 532 and 457 nm, when a series of peaks at 150–250 cm^–1^ gain considerable intensity, especially the one at 176 cm^–1^. Nine peaks observed in total in the Raman spectra match the number of Raman-active modes expected for the *C*_2*h*_ symmetry.

Linear-polarized Raman measurements (Figures 2, 3) allowed straightforward assignment of *A_g_* and *B_g_* types, as the former are active only in parallel configurations (*XX*, *YY*), and the latter only in cross-polarized configurations (*XY*, *YX*). Even the very weak feature at 166 cm^–1^ could be clearly assigned as the $B_{g}^{\left(2\right)}$ mode in the *XY*/*YX* polarized spectra. The clean separation of the two symmetry types in polarization measurements indicates that the structure is well described by the *C*_2*h*_ symmetry. Interestingly, while *XY* and *YX* configurations give identical intensity distribution for *B_g_* modes, the intensity of *A_g_* modes in *XX* and *YY* configurations changes considerably, particularly for the $A_{g}^{\left(3\right)}$ mode at 176 cm^–1^. Measurements with rotation of the laser polarization plane also show the high intensity of this peak only in the polarizer orientation parallel to *X* (Figure 3). Since this mode is resonantly enhanced when the spectrum is excited at 532 nm, its polarization behavior indicates that the resonant electronic excitation is polarized along the X direction, which coincides with the 2-fold symmetry axis.

Similar to RhSeCl, much better polarization properties were obtained after thinning down larger crystals using the Scotch tape technique. Interestingly, when using the method for RhTeCl, we found that the exfoliated flakes tended to have an elongated aspect ratio, attaining needle-like shapes after several iterations (Figure S2). A similar observation was also made by Köhler and Urland,^19^ who reported that the crystals could be mechanically split into fine fibers along the [010] axis, which is the direction of the symmetry axis in the *C*2/*m* group. This tendency to reduce the dimensionality not only by losing interlayer van der Waals contacts but also by breaking the layers along the directions with repeating anion types is encoded in the moniker “1.5-dimensional” used for this material in the title.

DFT-calculated frequencies of *A_g_* and *B_g_* modes show very good agreement with experimental values (Table 2). Four IR peaks can be attributed to *B_u_* modes, but the feature at 165 cm^–1^ has no calculated *B_u_* or *A_u_* counterparts and may be caused by the weakly activated $B_{g}^{\left(2\right)}$ mode. Neither of the two *A_u_* modes has sufficient IR intensity to be detected in the experimental spectra. Altogether, all nine Raman modes and four out of six IR-active modes of RhTeCl are identified.

**Table 2:** DFT-Computed Vibrational Frequencies (cm^–1^) of the Layer and Crystal RhTeCl and Their Comparison to Experimental Far-IR and Raman Spectroscopic Data^a^ ^b^

**DFT layer,** ***C***_**2** ***h***_		**DFT crystal,** ***C***_**2** ***h***_		**Exp. far-IR**		**exp. Raman**532/785**nm**		*χ* ^c^ **(cm** ^**–1**^ **/K)**
$B_{u}^{\left(1\right)}$	0	$B_{u}^{\left(1\right)}$	0					
$B_{u}^{\left(2\right)}$	0	$B_{u}^{\left(2\right)}$	0					
$A_{u}^{\left(1\right)}$	0	$A_{u}^{\left(1\right)}$	0					
$A_{g}^{\left(1\right)}$	80.4	$A_{g}^{\left(1\right)}$	81.7			81	w/w	–0.009(1)
$B_{g}^{\left(1\right)}$	98.9	$B_{g}^{\left(1\right)}$	97.7			99	s/vs	–0.008(1)
$A_{u}^{\left(2\right)}$	125.6	$A_{u}^{\left(2\right)}$	138.0					
$A_{g}^{\left(2\right)}$	142.5	$A_{g}^{\left(2\right)}$	146.4			151	m/w	–0.013(1)
$B_{g}^{\left(2\right)}$	146.3	$B_{u}^{\left(3\right)}$	151.2	149	s			
$B_{u}^{\left(3\right)}$	155.7	$B_{g}^{\left(2\right)}$	157.1	165	w	166	vw/–	
$A_{g}^{\left(3\right)}$	163.2	$A_{g}^{\left(3\right)}$	169.8			176	vs/w	–0.014(1)
$B_{u}^{\left(4\right)}$	183.7	$B_{u}^{\left(4\right)}$	192.6	203	ms			
$A_{g}^{\left(4\right)}$	192.3	$A_{g}^{\left(4\right)}$	199.9			208	w/vw	–0.016(1)
$B_{g}^{\left(3\right)}$	210.1	$A_{u}^{\left(3\right)}$	213.8					
$A_{u}^{\left(3\right)}$	210.9	$B_{g}^{\left(3\right)}$	214.8			221	m/w	–0.020(1)
$B_{u}^{\left(5\right)}$	216.4	$B_{u}^{\left(5\right)}$	216.9	221	w			
$A_{g}^{\left(5\right)}$	217.2	$A_{g}^{\left(5\right)}$	223.8			228	m/vw	–0.019(1)
$A_{g}^{\left(6\right)}$	240.4	$A_{g}^{\left(6\right)}$	242.5			249	m/–	–0.016(1)
$B_{u}^{\left(6\right)}$	244.4	$B_{u}^{\left(6\right)}$	246.1	252	m			

a DFT refers to the PBE functional and computations with unit cell parameters fixed to their experimental values.

b The relative intensity scale is as follows: vs – very strong, s – strong, m – medium, w – weak, vw – very weak.

c χ stands for the first-order temperature (FOT) coefficient, determined as the slope of a linear fit.

Vibrational displacements for eight Raman modes are shown in Figure 2d. Displacements of *A_g_* modes are perpendicular to the symmetry axis, while oscillations of *B_g_* modes are parallel to the layers. In terms of the main atomic contributions, $A_{g}^{\left(1\right)}$ at 83 cm^–1^ and $B_{g}^{\left(1\right)}$ at 99 cm^–1^ are combined oscillations of Rh and Te, $A_{g}^{\left(2\right)}$ at 151 cm^–1^ is largely a Te mode, $A_{g}^{\left(3\right)}$ at 176 cm^–1^ and $A_{g}^{\left(4\right)}$ at 208 cm^–1^ are mainly motions of Cl atoms, whereas $A_{g}^{\left(6\right)}$ is predominantly in-plane motion of Rh.

### Interlayer Interactions and Vibrational Modes

Interlayer interactions in 2D materials are mainly determined by van der Waals forces, but other components, such as electrostatic contributions, may also play a certain role. The ternary element composition of chalcohalides suggests that their charge distribution is less homogeneous than in binary chalcogenides or halides, which usually have identical chalcogenide or halide sublayers on both sides of the metal sublayer. The dominant role of Coulomb interaction in RhSeCl and RhTeCl follows from the weak sensitivity of the DFT results to the presence of dispersion correction in DFT calculations, as discussed above. In chalcohalides, the different electronegativity of nonmetals may play a role by itself, in addition to the unavoidable asymmetry in the arrangement of atoms around the metal sublayer. A simple insight into the charge distribution is given by Bader atomic charges listed in Table 3. While the chloride ion bears similar charges of −0.46 in RhSeCl and −0.49 in RhTeCl, a different charge balance is found in the Rh–Se and Rh–Te pairs. The higher electronegativity of Se implies that it should be less positively charged than Rh, and we indeed find almost noncharged Se (+0.05) and Rh with a positive charge of +0.41. In RhTeCl, it is Te, which has lower electronegativity, resulting in its positive charge of +0.47, while Rh bears a negligible charge of +0.02. Thus, for the Janus RhSeCl layer, we have the Cl-side with a considerable negative charge and the noncharged Se-side, whereas in RhTeCl, we can expect an alternation of negatively charged Cl and positively charged Te stripes on each side of the layer. While atomic charges have no unique definition, and the peculiarities of a given charge partition scheme between the atoms can be questioned, the electrostatic potential imposed around the atoms is uniquely defined from the electron density and is a more universal indicator. The electrostatic potential mapped on the electron density isosurfaces of RhSeCl and RhTeCl layers in Figure 4 fully supports the picture derived from Bader atomic charges. For a stack of layers, this charge distribution leads to the accumulation of the dipole moment in RhSeCl with each additional layer. In RhTeCl, on the other hand, closely located opposite charges will partially compensate each other in interlayer interactions, while inversion symmetry ensures the complete cancellation of the overall dipole moment.

**Table 3:** Pauli Electronegativity and Bader Charges (Q) in RhSeCl and RhTeCl

	Electronegativity	Q in RhSeCl	Q in RhTeCl
Rh	2.28	+0.41	+0.02
Se	2.55	+0.05	
Te	2.10		+0.47
Cl	3.16	–0.46	–0.49

**Figure 4:**
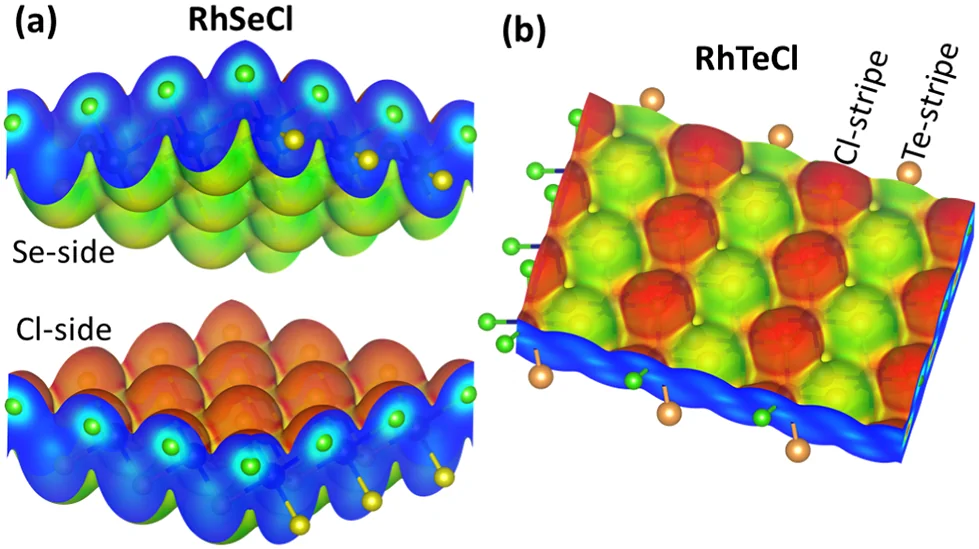
Electrostatic potential mapped on the electron density isosurface of 0.01 au in (a) the RhSeCl layer, with two sides shown separately; (b) the RhTeCl layer, where only one side is shown since the other one has the same pattern. Red and green colors correspond to more and less negative potential, respectively.

In the lattice dynamics of van der Waals materials, interlayer interactions are reflected in two related phenomena: (i) changes in the optical phonons under the influence of the interactions, which can be evaluated by comparing the modes of an isolated layer and the crystal; (ii) rigid-layer shear and breathing modes (interlayer modes). In RhSeCl, each optical mode of the isolated layer gives rise to two crystal modes, and their splitting, which in essence is nothing more than the Davydov splitting, can be used as an indicator of the interlayer interactions. The *E*_1_/*E*_2_ pairs observed at 179/176 cm^–1^ and 270/272 cm^–1^ demonstrate that the splitting of the Γ-point optical phonons of the single RhSeCl layer in the crystal is small. Theory similarly predicts the splitting in *E*_1_/*E*_2_ and *A*_1_/*B*_1_ pairs by no more than 7 cm^–1^, and often less, altogether suggesting that interlayer interactions in RhSeCl are not very strong.

Further information on the strength of interlayer interactions can be obtained from the analysis of the interlayer vibrational modes.^37,38^ The shear mode of RhSeCl, $E_{2}^{\left(1\right)}$ , is detected at 26 cm^–1^ (DFT calculated at 25.4 cm^–1^), whereas the calculated frequency of the breathing mode $B_{1}^{\left(1\right)}$ is 49.1 cm^–1^. Direct comparison of interlayer mode frequencies in various layered compounds is not meaningful because of the different atomic masses, but a reasonable analysis can be done for interlayer force constants, which, in a linear chain model, can be calculated from corresponding vibrational frequencies (*ω*) as *K* = *μ*(*πcω*)^2^, where *μ* is the mass per unit area of a given layer, and *c* is the speed of light.^38^ Table 4 lists shear (*ω^S^*) and layer breathing (*ω^LB^*) vibrational frequencies and derived parameters for selected van der Waals materials based on the data from refs 37 and 38. The shear force constant *K^S^* of RhSeCl, 2 × 10^19^ N m^–3^ is larger than that of graphite (1.28 × 10^19^ N m^–3^) and h-BN (1.83 × 10^19^ N m^–3^), which have the lowest reported values among van der Waals materials, but it is considerably smaller than that in transition metal chalcogenides (2.3–4.6 × 10^19^ N m^–3^). The interlayer breathing force constant *K^LB^* = 7.3 × 10^19^ N m^–3^ is also lower in RhSeCl than in typical M^III^-chalcogenides (∼9 × 10^19^ N m^–3^) with the exception of Bi_2_Se_3_, which has an anomalously small *K^LB^*.

**Table 4:** Interlayer Modes and Derived Parameters in Selected van der Waals Materials^a^ ^b^

	*ω^S^* (cm^–1^)	*ω^LB^* (cm^–1^)	*K^S^* (10^19^ N m^–3^)	*K^LB^* (10^19^ N m^–3^)	Ref.
RhSeCl	26 (25.4)	(49.1)	1.96 (2.05)	(7.33)	t.w.
RhTeCl	(23.9/26.9)	(39.5)	(1.85_c_/2.35_a_)	(5.06)	t.w.
graphite	43.5	128 (INS)	1.28	11.1	^37,38^
*h*-BN	52.2	121 (IXS)	1.83	9.83	^37,38^
black P	19.4/51.6 (INS)	87.1 (INS)	0.61/3.3	12.7	^38^
MoS_2_	32.5	56.8 (LCM)	2.82	8.90	^37,38^
MoSe_2_	25.5	48.1 (LCM)	2.60	9.25	^37,38^
MoTe_2_	26.8	39.3 (LCM)	4.25	9.12	^38^
WS_2_	27.5	47.8 (LCM)	3.16	9.55	^37,38^
WSe_2_	23.8	40.3 (LCM)	3.06	8.77	^37,38^
Bi_2_Se_3_	18.0 (LCM)	29.3 (LCM)	2.27	5.26	^41^
Bi_2_Te_3_	25.4 (LCM)	42.4 (LCM)	4.57	13.33	^41^
MoCl_3_	(41.8/43.8)	(67.4)	(3.62/3.98)	(9.41)	^40^
RuCl_3_	20 (THz)	48 (THz)	0.80	4.63	^39^

a The values in the table are mainly based on compilations from refs 37 and 38.

b When corresponding vibrations are not Raman-active for bulk van der Waals material, *ω^S^* and particularly *ω^LB^* frequencies are determined by other techniques: LCM – linear chain model fitting for few-layer samples, INS – inelastic neutron scattering, IXS – inelastic X-ray scattering, THz – THz spectroscopy. Frequencies in parentheses are based on DFT calculations.

Γ-point vibrations of a single layer and a crystal of RhTeCl have similar frequencies, the largest deviation being 9 cm^–1^, but mainly less than 5 cm^–1^. As in RhSeCl, this modest influence of the layer stacking on vibrational modes points to the weak interlayer interactions. Interlayer modes of RhTeCl do not appear at the Γ-point and hence are not accessible in IR and Raman spectra, but they become available at the Y-point (Figure S3), with calculated frequencies of 23.9 and 26.9 cm^–1^ for the two components of the shear mode, and 39.5 cm^–1^ for *ω^LB^*. Transformation to force constants gives 1.85/2.35 × 10^19^ N m^–3^ for *K^S^*, quite close to the values in RhSeCl, and 5.06 × 10^19^ N m^–3^ for *K^LB^*, which is considerably smaller.

The comparison of interlayer force constants cannot be complete without 2D halides, but the literature data on their interlayer force constants are very scarce, mainly because their interlayer modes do not appear in Raman spectra. For RuCl_3_, *ω^S^* of 20 cm^–1^ and *ω^LB^* of 48 cm^–1^ were estimated by THz spectroscopy,^39^ giving *K^S^* of 0.80 × 10^19^ N m^–3^ and *K^LB^* of 4.63 × 10^19^ N m^–3^. However, this assignment may be questionable since MCl_3_ has no shear and breathing modes at the Γ-point, similar to RhTeCl. For the recently studied MoCl_3_,^40^ our DFT calculations produce *ω^S^* of 41.8/43.8 cm^–1^ and *ω^LB^* of 67.4 cm^–1^ in Y-point, resulting in the *K* *^S^* and *K^LB^* values of 3.62/3.98 × 10^19^ N m^–3^ and 9.41 × 10^19^ N m^–3^, respectively. The spread between RuCl_3_ and MoCl_3_ is too large, and more data will be needed on this class of layered materials for reliable analysis.

### Raman Spectra at Different Excitation Energies

Variation of the Raman spectra with the excitation wavelength, as shown in Figure 1 suggests the presence of resonance effects^42,43^ and prompts a more detailed study. We therefore used a dye laser charged with rhodamine 6G or 2-methyl-6-(4-dimethylaminostyryl)-4H-pyran (DCM) and tuned the excitation wavelength between 570 and 720 nm in 10 nm steps. The evolution of the spectra measured with a gradual variation of the excitation energy is shown in Figure 5a,b, while excitation profiles for selected peaks are plotted in Figure 6.

**Figure 5:**
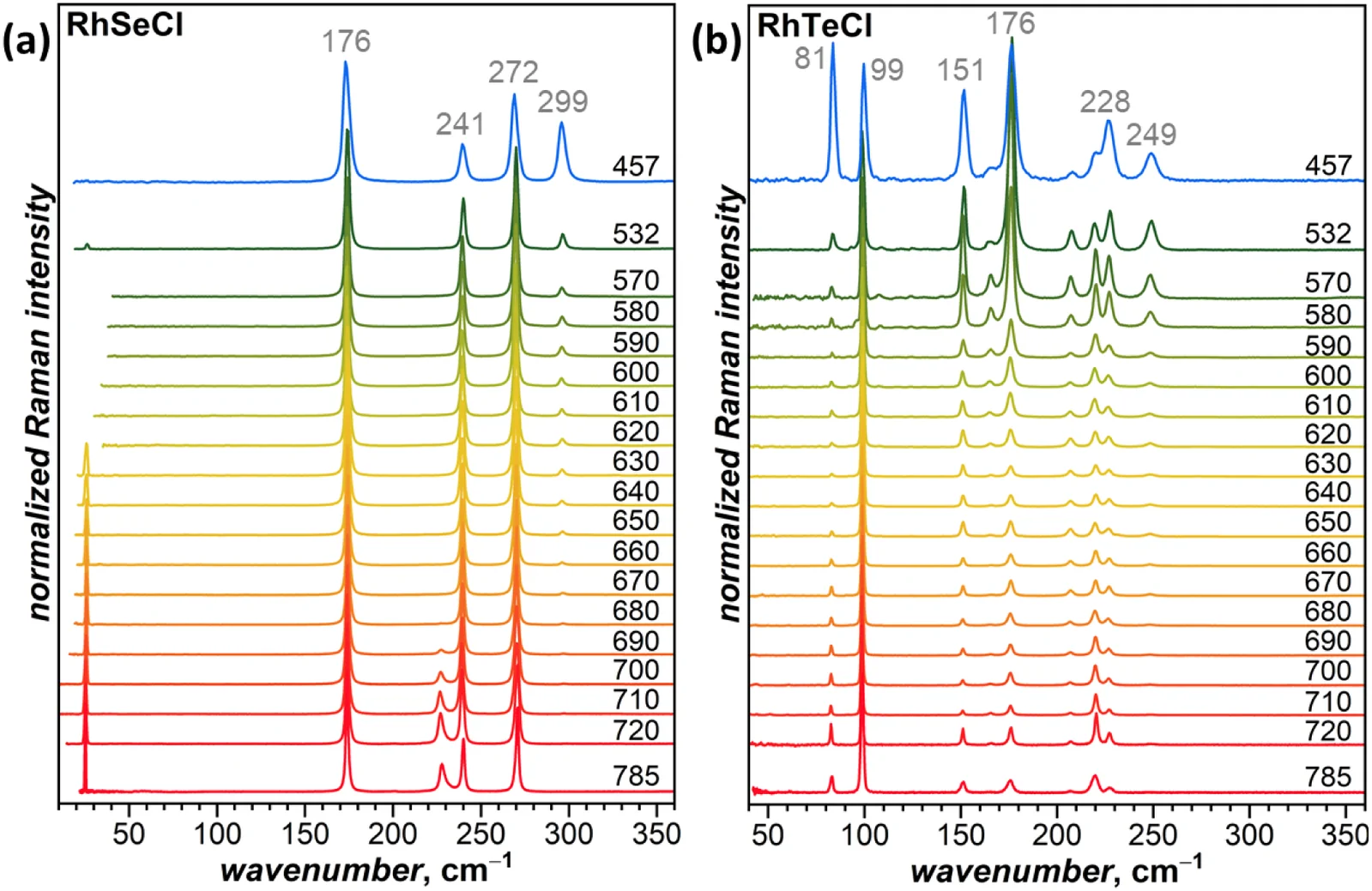
(a, b) Room-temperature Raman spectra of (a) RhSeCl crystals and (b) RhTeCl crystals measured with different excitation laser lines (laser wavelength is listed for each curve). Spectra are normalized to the intensity of the mode at 176 cm^–1^ for RhSeCl and mode the mode at 99 cm^–1^ for RhTeCl. In (a), the low-frequency part of the spectra could not be measured with excitation at 620–570 nm because of the broader tail of the excitation laser.

**Figure 6:**
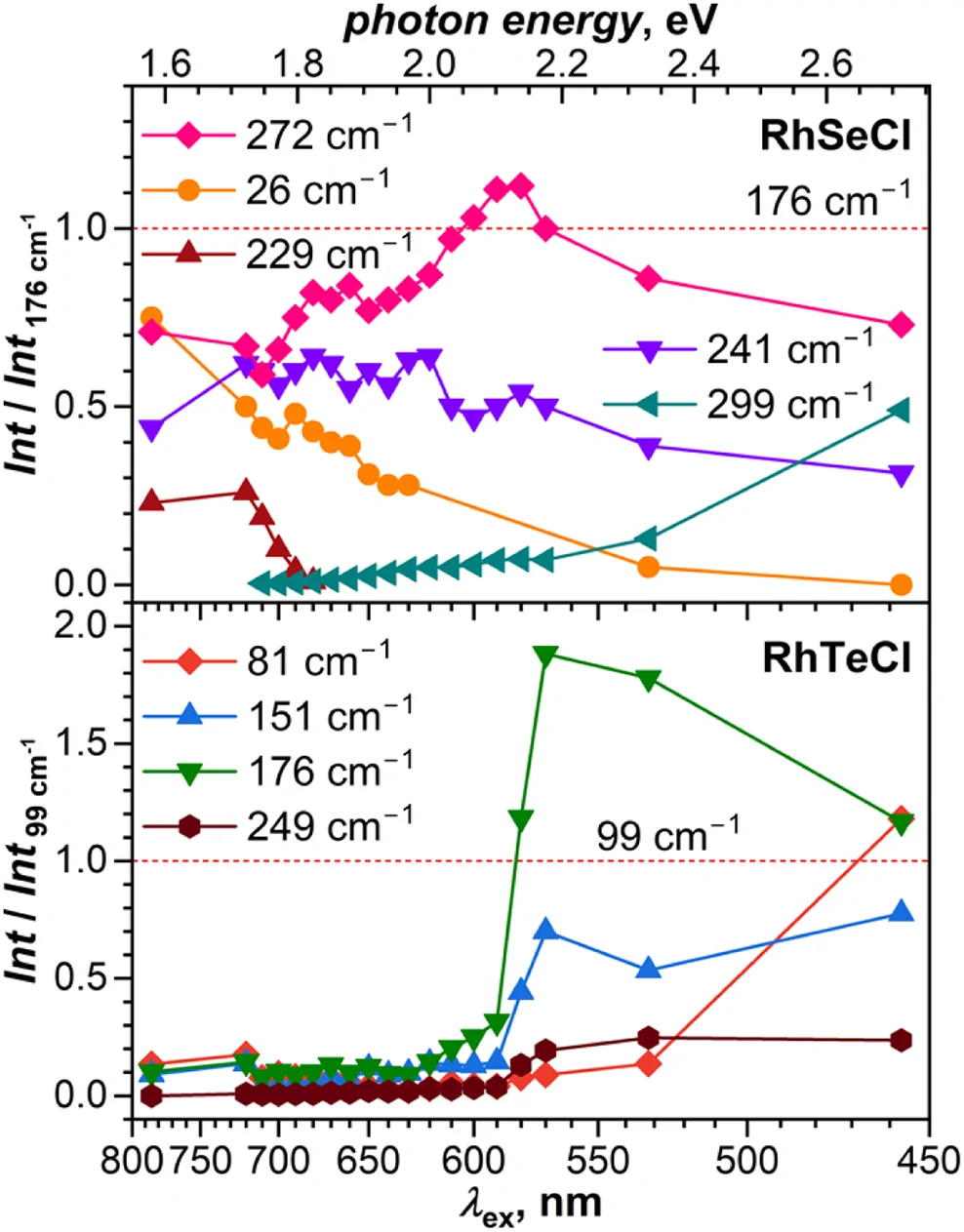
Excitation profiles for selected Raman modes are shown relative to the $A_{1}^{\left(2\right)}$ mode at 176 cm^–1^ (RhSeCl) and the $B_{g}^{\left(1\right)}$ mode at 99 cm^–1^ (RhTeCl).

For RhSeCl, $E_{2}^{\left(2\right)}$ , $A_{1}^{\left(2\right)}$ , and $E_{2}^{\left(3\right)}$ modes at 176, 241, and 272 cm^–1^ retain their high intensity throughout the entire excitation range. Relative to $E_{2}^{\left(2\right)}$ , intensity of $E_{2}^{\left(3\right)}$ reaches its maximum at 580–590 nm, but the ratio varies by less than a factor of 2. The $A_{1}^{\left(2\right)}$ / $E_{2}^{\left(2\right)}$ ratio varies even less. The main changes occur with the peaks at 26, 229, and 299 cm^–1^. The shear mode at 26 cm^–1^, which has the second highest peak at 785 nm (75% relative to 176 cm^–1^), steadily decreases intensity with the increase of the excitation energy, reducing to 28% at 630 nm and to only 5% at 532 nm. Likewise, the combinational mode at 229 cm^–1^ has the highest intensity at 785 and 720 nm, and then decays fast with the shortening of the excitation wavelength until it cannot be detected at all at λ_ex_ < 680 nm. Conversely, the $A_{1}^{\left(3\right)}$ mode at 299 cm^–1^, which is barely visible at 710 nm (less than 1% of the peak at 176 cm^–1^), gradually increases its relative intensity to 13% at 532 nm and 49% at 457 nm.

In the spectra of RhTeCl, the changes with excitation wavelength happen more abruptly. The spectra with the strongly dominant $B_{g}^{\left(1\right)}$ mode at 99 cm^–1^ remain almost unchanged between 785 and 620 nm. Starting at 610 nm, the relative intensities of *A_g_* modes at 151 cm^–1^ $\left(A_{g}^{\left(2\right)}\right)$ , 228 cm^–1^ $\left(A_{g}^{\left(5\right)}\right)$ , 249 cm^–1^ $\left(A_{g}^{\left(6\right)}\right)$ , and especially $A_{g}^{\left(3\right)}$ at 176 cm^–1^ start to grow. The most pronounced changes occur between 590 and 570 nm. Relative to $B_{g}^{\left(1\right)}$ , the intensity of $A_{g}^{\left(3\right)}$ grows from 14% at 620 nm to 32% at 590 nm and reaches its highest value of 188% at 570 nm. At shorter wavelengths, the relative intensity remains almost equally high (178%) at 532 nm, and then decreases to 117% at 457 nm. The maximum intensity of the $A_{g}^{\left(3\right)}$ mode probably occurs between 570 and 532 nm, but this excitation range is not accessible with our setup. Another remarkable development at short excitation wavelengths is the strong enhancement of the $A_{g}^{\left(1\right)}$ mode at 81 cm^–1^ from 14% at 532 nm to 118% at 457 nm.

Electronic spectra of RhSeCl and RhTeCl were probed using diffuse reflectance spectroscopy of powder samples. To estimate absorption spectra from diffuse reflectance, the Kubelka–Munk transformation was applied (abbreviated as KM-*R*). Figure 7 presents the results of reflectance measurements for both compounds compared to low-temperature photoluminescence (PL) spectra, which provide a lower bound for the lowest-energy optical transitions. Earlier DFT and EELS studies suggested that both RhTeCl and RhSeCl are semiconductors with indirect bandgaps of ∼0.8 and ∼1.4 eV, respectively.^20^ The diffuse reflectance spectra of both compounds are not highly structured and show only a gradual increase in absorption intensity toward higher energies. Raman excitation profiles appear to be more sensitive to variations in the electronic structure than direct measurements of electronic excitations.

**Figure 7:**
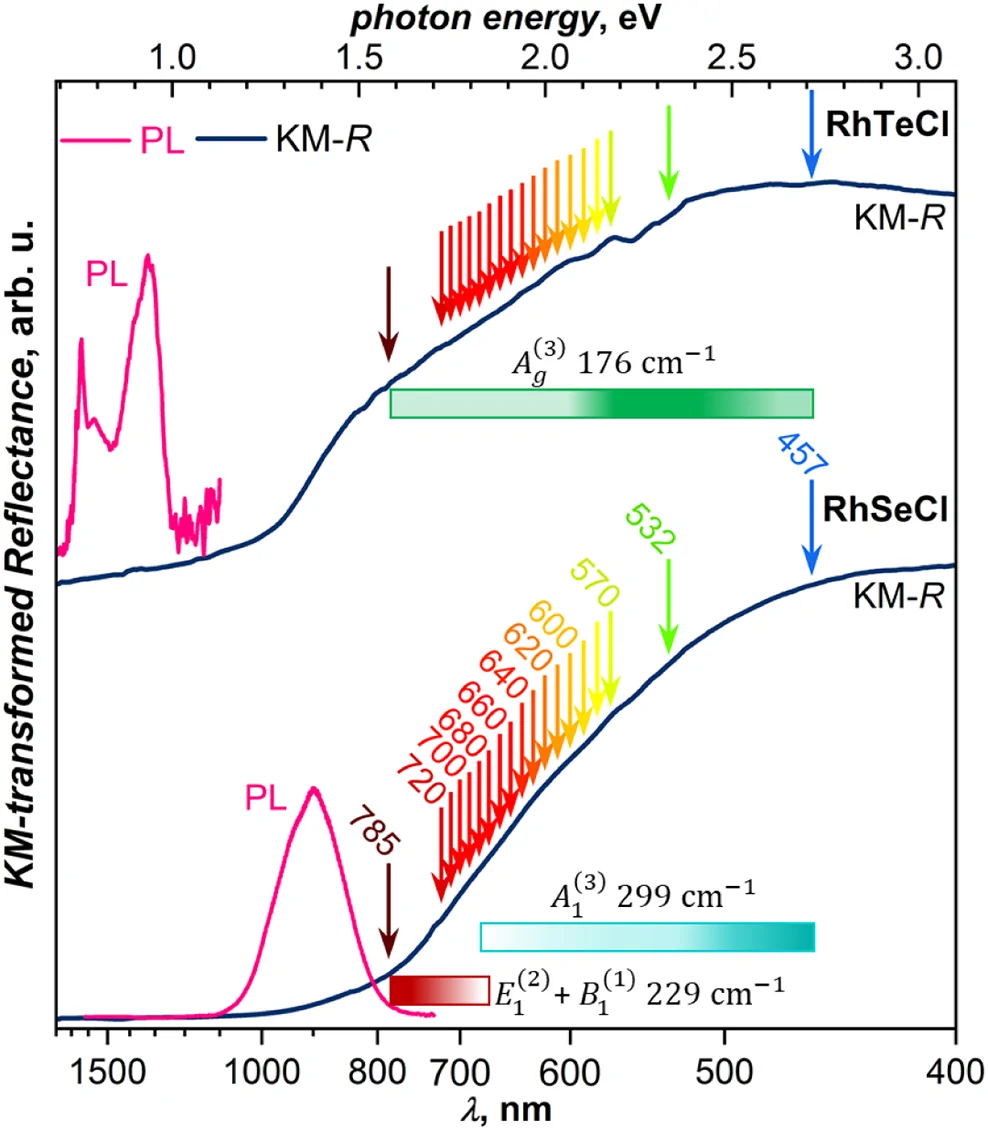
Optical spectra of RhTeCl and RhSeCl: photoluminescence spectra (pink, PL) and diffuse-reflectance spectra after the Kubelka–Munk transformation (dark blue, KM-*R*). PL spectra were measured for crystals at 5 K, while reflectance spectra were measured at room temperature for powder samples. Arrows denote the positions of laser lines used in Raman measurements (see Figure 5). Pronounced resonant Raman enhancement of selected vibrational modes is shown with gradient-filled rectangles.

### Temperature Dependence of Raman Spectra

Variable-temperature Raman measurements of RhSeCl and RhTeCl were performed with excitation at 532 nm in the temperature range of 10–300 K (Figure 8a,b). Neither compound exhibits splitting of the peaks or the appearance of new features upon cooling. Instead, the spectra show gradual shifts in peak positions and some variations in intensities and line widths. Variable-temperature peak positions and widths determined from Lorentzian peak fitting are plotted for selected modes in Figure 8c,d (data for other modes can be found in Supporting Information, Figures S4 and S5). The temperature dependence of the mode frequencies is not very smooth and may be affected by structural variations, as indicated by abrupt changes in the line width dependence (*vide infra*). For this reason, a reliable description of the data, including temperature terms of different orders and various mechanisms of phonon–phonon interactions, such as those developed by Balkanski et al.,^44^ appears not possible at this moment. Instead, we use here a simple linear approximation, ν­(*T*) = ν_0_ + *χT*, where χ is interpreted as an empirical first-order temperature (FOT) coefficient, which is used for comparison between RhSeCl and RhTeCl and other van der Waals materials without delving into the details of exact mechanisms.

**Figure 8:**
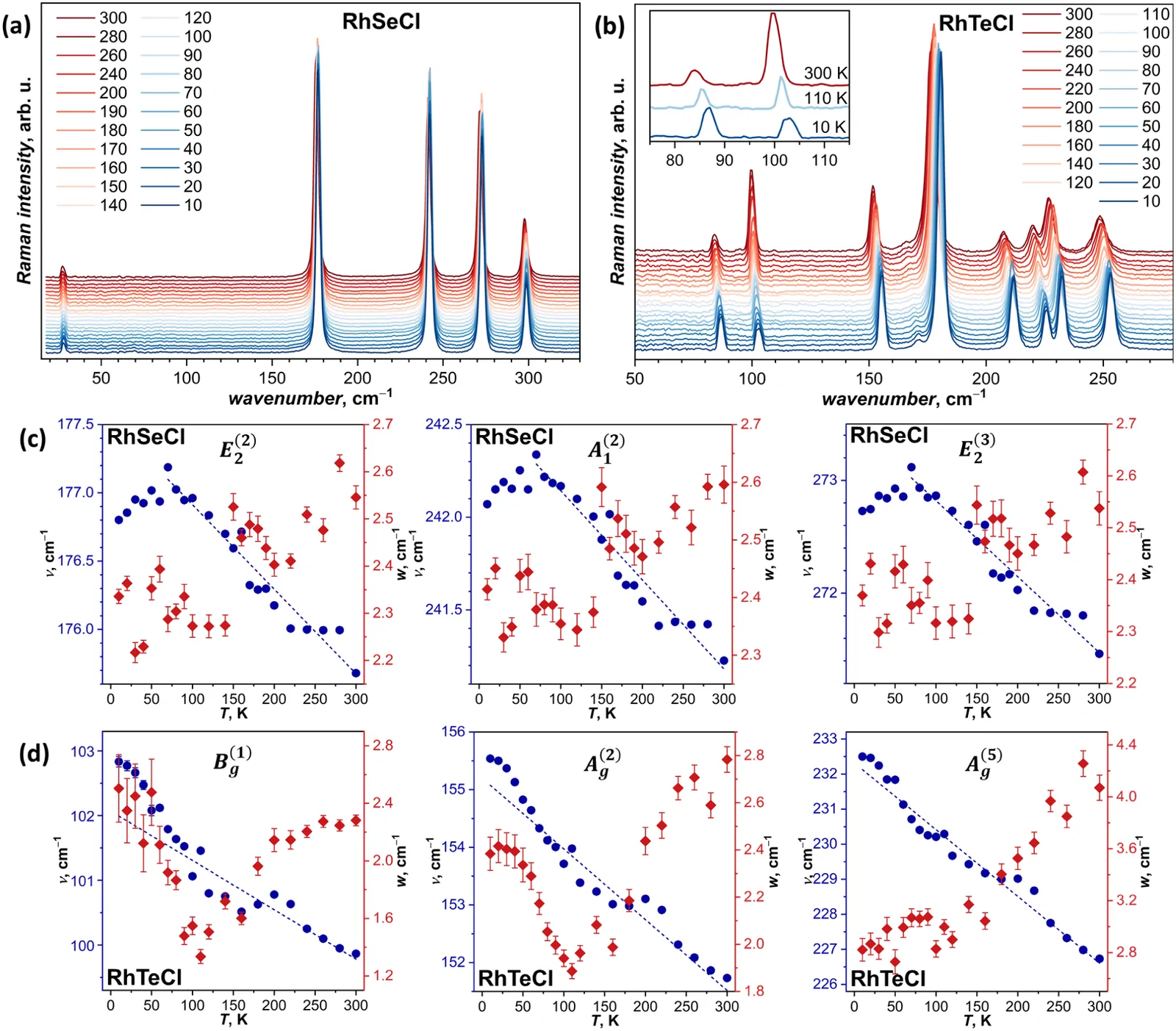
(a, b) Variable-temperature Raman spectra of (a) RhSeCl and (b) RhTeCl measured between 10 and 300 K, with excitation at 532 nm. (c, d) Temperature variation of the peak maximum (ν) and the half-width at half-maximum (w) for selected vibrational modes of (c) RhSeCl and (d) RhTeCl; dashed lines represent linear fits of peak position versus temperature. See the Supporting Information for analogous graphs for other modes.

Raman spectra of RhSeCl show comparably weak temperature dependence. Between 10 and 70 K, vibrational frequencies increase slightly, but the total change in this interval is less than 0.3 cm^–1^. Above 70 K, the modes steadily soften with the total frequency shift of ∼1.5 cm^–1^ between 70 and 300 K. This behavior is typical for optical phonons and is caused by anharmonic phonon–phonon interactions and thermal expansion.^45^ FOT coefficients χ for RhSeCl modes are found in the range from −0.005 to −0.007 cm^–1^ K^–1^ (Table 1), which is smaller than the values of −0.007 to −0.017 cm^–1^ K^–1^ typically observed for transition metal dichalcogenides.^45^ Line widths of RhSeCl optical phonons show only moderate changes with temperature and vary from 2.2–2.3 cm^–1^ at 10 K to 2.6–2.7 cm^–1^ near room temperature. Nonetheless, one can notice a jump in the peak width of all modes at 150 K, which has no counterparts in peak positions (Figure 8c).

Temperature dependence of peak positions and line widths of RhTeCl is more pronounced, with the frequency shifts in the whole temperature range reaching 6 cm^–1^. The span of FOT coefficients from −0.008 to −0.020 cm^–1^ K^–1^ (Table 2) is two to three times larger than that found for RhSeCl. Peak widths of RhSeCl change with temperature nonmonotonously, which is especially well seen for the $A_{g}^{\left(2\right)}$ mode (Figure 8d). Its width drops from 2.4 to 1.8 cm^–1^ between 10 and 110 K, but then starts to grow at higher temperatures, reaching 2.8 cm^–1^ at 300 K. Such temperature dependence may be an indication of a phase transition. A similar peculiarity at 110 K is found for other modes below 200 cm^–1^ (see the SI), but it is less visible for vibrations at higher frequencies, as exemplified by the $A_{g}^{\left(5\right)}$ mode in Figure 8d. Relative intensities of RhTeCl also demonstrate considerable temperature dependence, as best illustrated by the range of 70–120 cm^–1^ show in the inset in Figure 7b. Between 10 and 300 K, the intensity ratio of $A_{g}^{\left(1\right)}$ and $B_{g}^{\left(1\right)}$ modes changes from 1:0.7 to 1:4.3. Considerable temperature variations of relative intensities are also observed in the frequency range of 200–260 cm^–1^.

## Conclusions

Comprehensive spectroscopic study of the lattice dynamics in two-layered rhodium chalcocylides, RhSeCl and RhTeCl, allowed detailed assignment of all their Raman-active and some IR-active vibrations. Excellent agreement between DFT calculations and experimental data not only supports experimental assignments of fundamental modes based on polarized Raman measurements but also demonstrates that results of DFT calculations can be reliably used for experimentally inaccessible vibrations and for the complete phonon band structure. Knowing the phonon structure of layered materials is important for many reasons, including the use of Raman spectroscopy as an express analytical method for newly synthesized crystals and exfoliated flakes of 2D materials, the relation between rigid-layer modes and interlayer interactions, the relation to electronic structure through electron–phonon coupling, and even information on heat transfer properties. In this work, we particularly focused on the interplay between electronic excitations and Raman activity, for which we studied Raman spectra of RhSeCl and RhTeCl using multiple excitation energies in the visible part of the spectrum. Gradual variation of the excitation energy enabled by the dye laser demonstrated a strong sensitivity of some modes to the Raman excitation energy. Excitation profiles obtained after analysis of intensity variations at different excitation energies revealed hidden transitions with strong phonon–exciton coupling, which could not be resolved in reflectance spectra. Furthermore, variable-temperature Raman measurements revealed considerably different degrees of anharmonicity in the two materials and pointed to possible phase transitions at low temperatures.

## Supplementary Material



## Notes

Optical images of RhSeCl and RhTeCl crystals, computed phonon band structures, and additional variable-temperature Raman data (PDF)
