# Supplementary material for: Lattice Dynamics of van der Waals Layered Rhodium Chalcochlorides: Janus RhSeCl and 1.5-Dimensional RhTeCl
Source: Inorg Chem. 2026 Aug 10;65(33):19169–81. doi: 10.1021/acs.inorgchem.6c02052 (PMC13509008; doi:10.1021/acs.inorgchem.6c02052)
Supplement: Supplementary file 1 [file ic6c02052_si_001.pdf]

Supporting Information

Lattice Dynamics of van der Waals Layered Rhodium Chalcochlorides: Janus RhSeCl and 1.5-Dimensional RhTeCl

Sandra Schiemenz, Domenic Nowak, Samuel Froeschke, Marco Naumann, Marco Rosenkranz, Bernd Büchner, Martin Knupfer, Nico Gräßler, Silke Hampel, Stanislav M. Avdoshenko, Alexey A. Popov*

Leibniz Institute for Solid State and Materials Research Dresden (IFW Dresden), 01069 Dresden, Germany

*E-mail: a.popov@ifw-dresden.de

Temperature profile in the synthesis of RhSeCl	S2
Optical images of RhSeCl crystal and exfoliation products	S3
DFT-computed structures and vibrational frequencies	S4
Phonon band structure	S6
Variable-temperature Raman measurements	S7

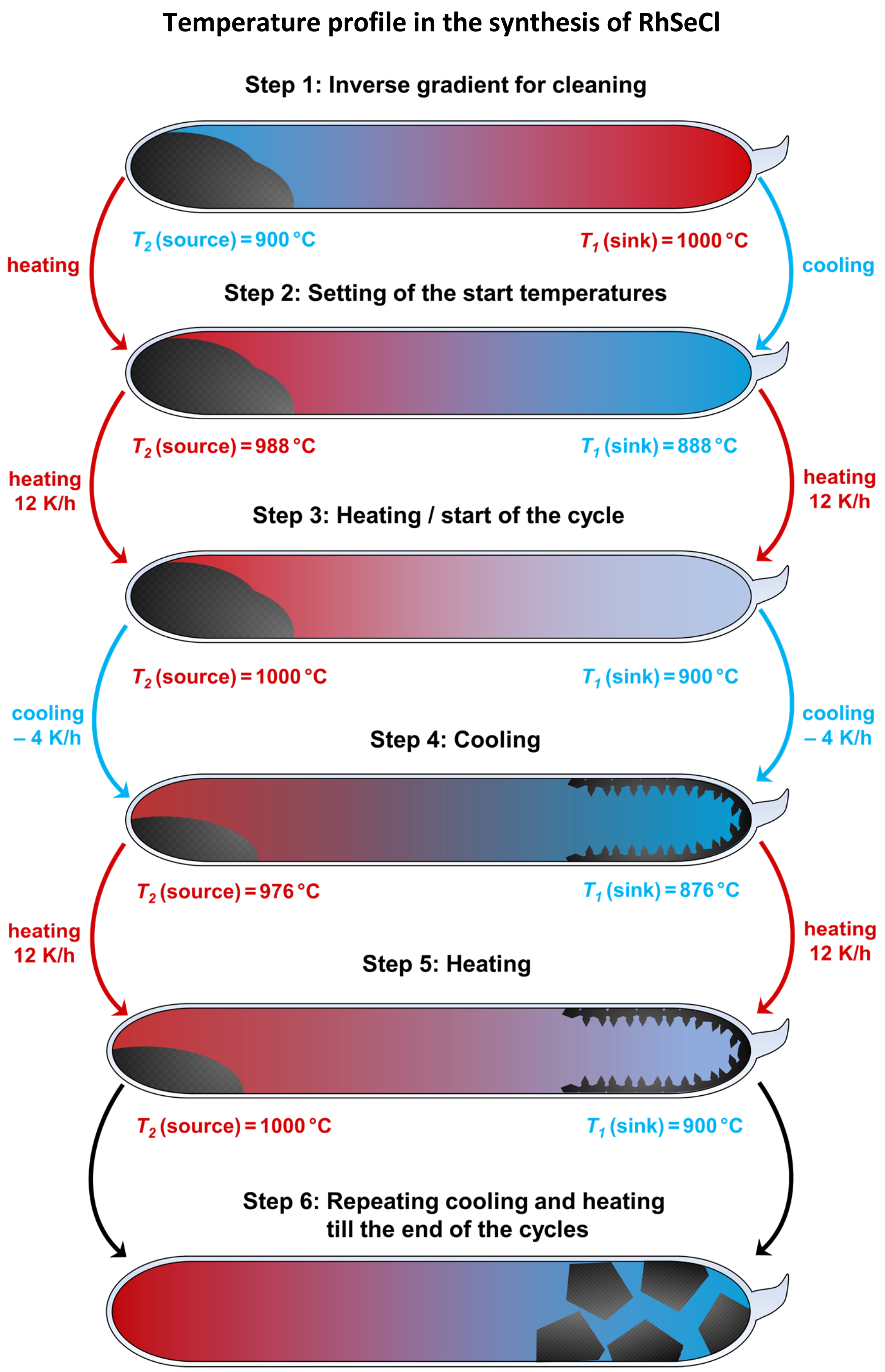

Figure S1. Schematic description of the temperature profile for the growth of RhSeCl crystals by temperature-oscillating chemical vapor transport method.

Optical images of RhSeCl crystal and exfoliation products

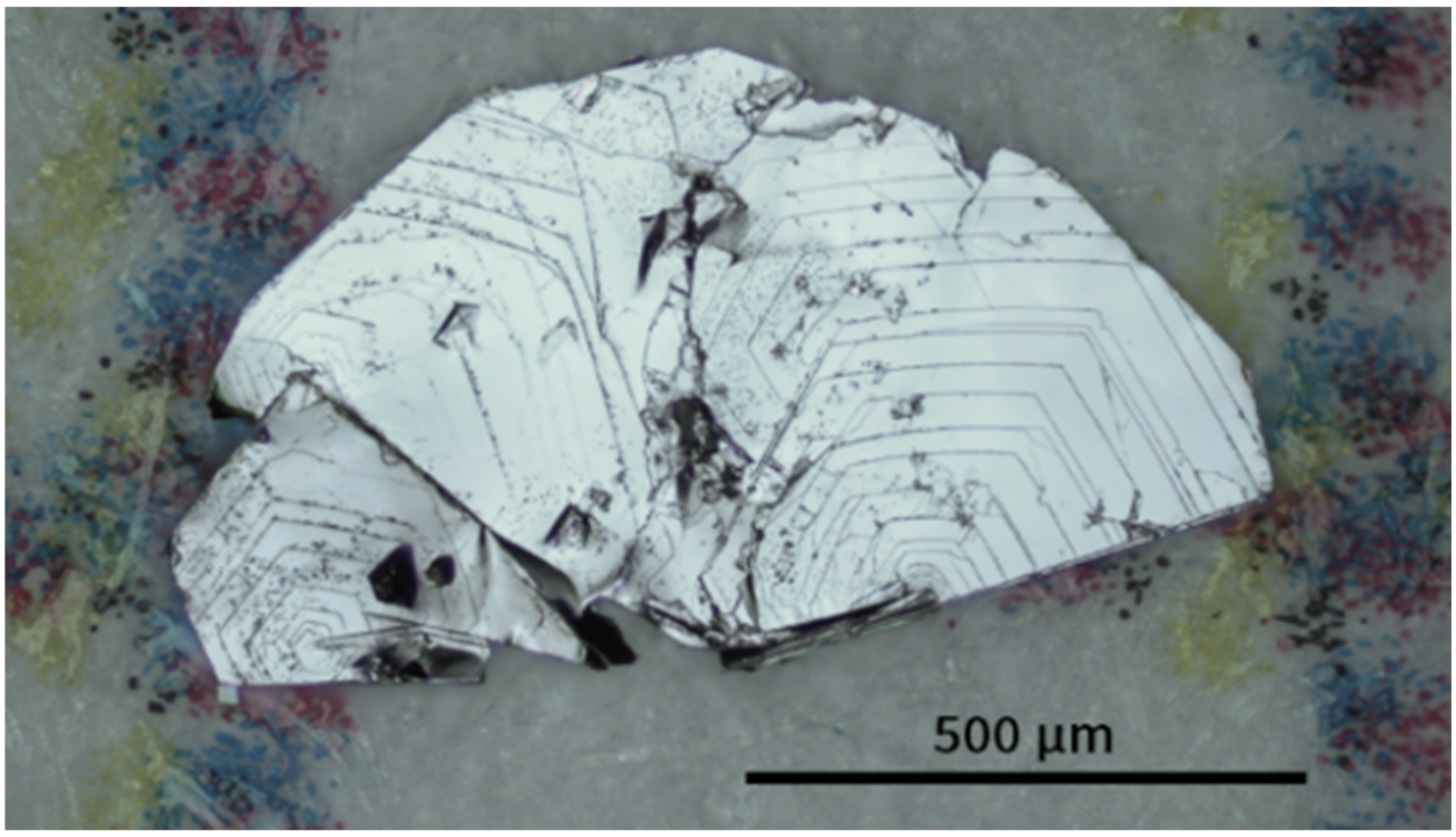

Figure S2. Optical image of a RhSeCl crystal obtained from temperature-oscillating chemical vapor transport.

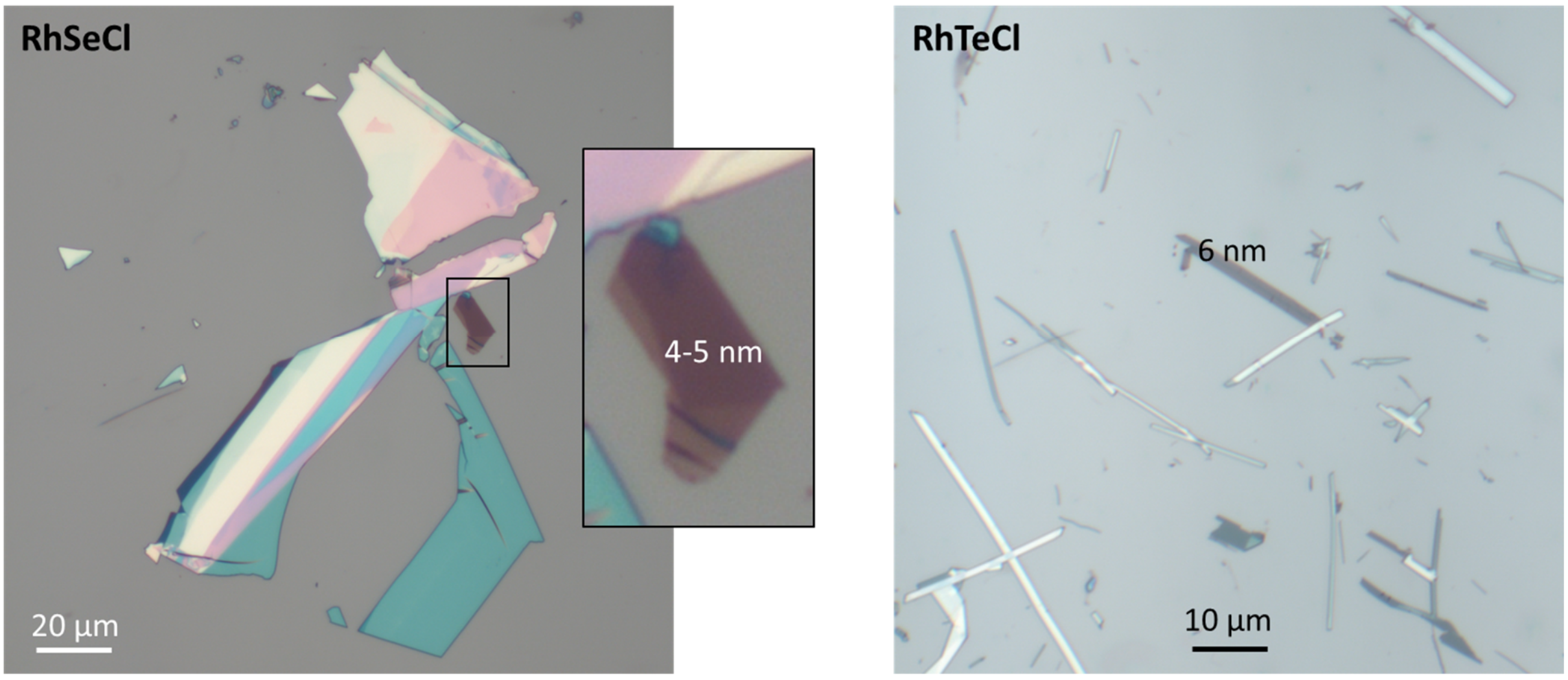

Figure S3. Typical shapes of microcrystalline flakes obtained after several iterations of scotch-tape exfoliation of RhSeCl and RhTeCl crystals. Thickness determined by AFM is shown for some crystals. While flakes of RhSeCl have irregular laterally extended shapes, RhTeCl tends to form long narrow fibers.

DFT-computed structures and vibrational frequencies

Table S1. Cell parameters (Å), Rh–Se and Rh–Cl bond lengths (Å), and vibrational frequencies (cm^{-1}) of RhSeCl from experiment and different computational models.

	exp.	PBE, exp. cell	PBE-D3, exp. cell	PBE, opt. cell	PBE-D3, opt. cell
a	3.488	3.488	3.488	3.534	3.529
c	11.579	11.579	11.579	11.612	11.417
d(Rh–Se)	2.401	2.399	2.400	2.423	2.410
d(Rh–Cl)	2.502	2.533	2.538	2.557	2.544
$E_2^{(1)}$	26	25.4	27.7	26.9	29.0
$B_1^{(1)}$		49.1	50.2	47.8	51.3
$E_2^{(2)}$	176	163.6	161.1	153.4	159.5
$E_1^{(2)}$	179	164.6	162.5	154.7	161.0
$A_1^{(2)}$	241	219.7	222.8	213.0	218.3
$B_1^{(2)}$		226.1	226.5	217.3	221.6
$E_1^{(3)}$	270	264.0	262.2	249.4	257.3
$E_2^{(3)}$	272	264.9	263.2	250.4	258.4
$A_1^{(3)}$	299	301.7	301.0	290.0	297.3
$B_1^{(3)}$		304.4	304.5	293.2	300.7

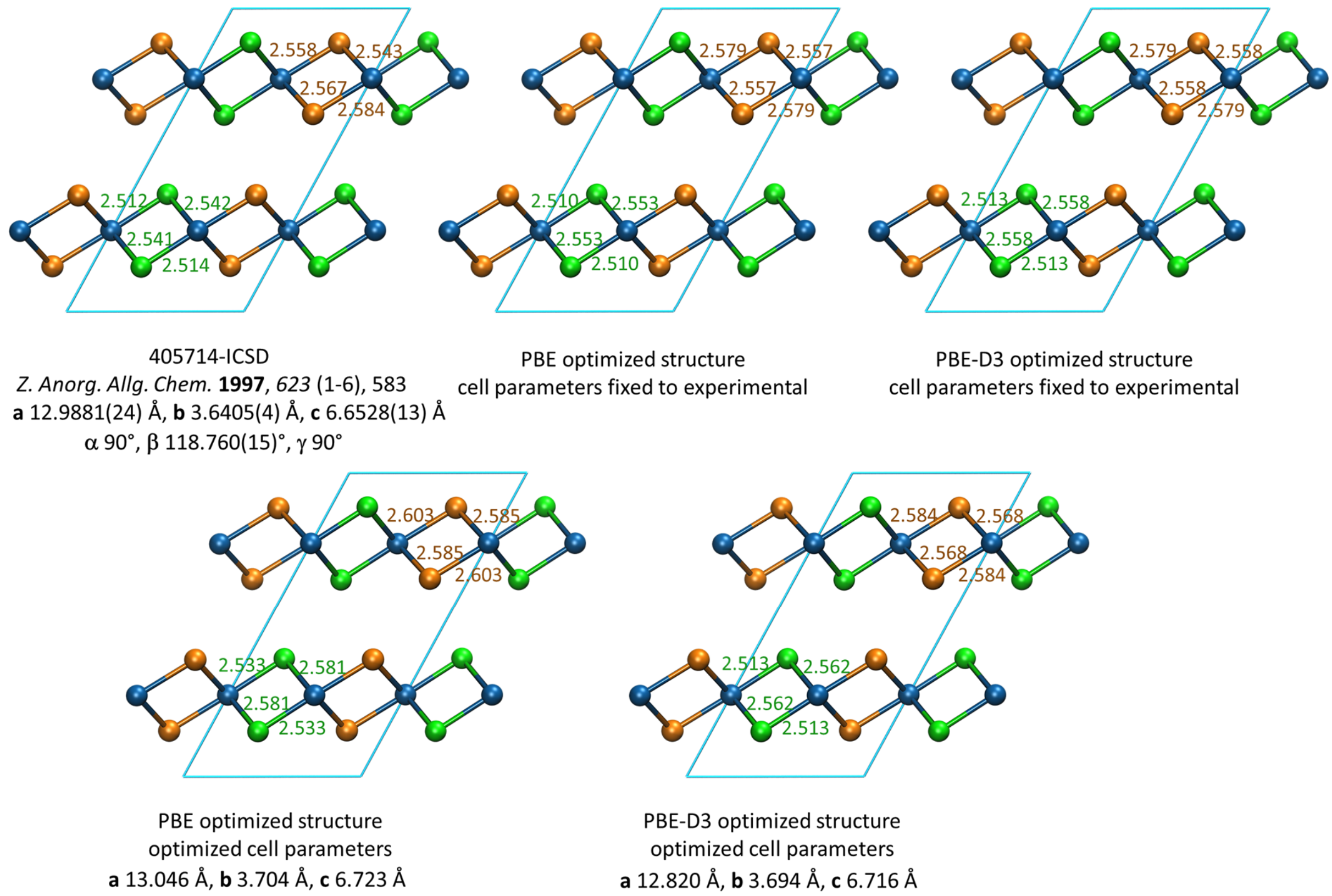

Figure S4. Comparison of Rh–Te (dark orange) and Rh–Cl (green) bond lengths in experimental crystal structure of RhTeCl (ICSD-405714) to results of DFT calculations with PBE and PBE-D3 functionals with and without optimization of the unit cell parameters.

Table S2. Cell parameters (Å), Rh–Te and Rh–Cl bond length (Å), and vibrational frequencies (cm^{-1}) of RhTeCl from experiment and different computational models.

	exp.	PBE, exp. cell	PBE-D3, exp. cell	PBE, opt. cell	PBE-D3, opt. cell
a	12.988	12.988	12.988	13.046	12.820
b	3.641	3.641	3.641	3.704	3.694
c	6.653	6.653	6.653	6.723	6.716
d(Rh–Te)	2.588/2.584	2.579	2.579	2.603	2.584
	2.567/2.543	2.557	2.558	2.585	2.568
d(Rh–Cl)	2.512/2.514	2.510	2.513	2.533	2.513
	2.541/2.542	2.553	2.558	2.581	2.562
$A_g^{(1)}$	81	81.7	82.6	83.9	82.8
$B_g^{(1)}$	99	97.7	98.2	97.5	98.7
$A_u^{(2)}$		138.0	137.9	128.4	136.0
$A_g^{(2)}$	151	146.4	145.1	140.5	147.2
$B_u^{(3)}$	149	151.2	151.3	144.0	147.2
$B_g^{(2)}$	166	157.1	156.7	149.7	159.2
$A_g^{(3)}$	176	169.8	171.4	165.7	171.6
$B_u^{(4)}$	203	192.6	196.5	186.0	194.5
$A_g^{(4)}$	208	199.9	202.0	193.4	201.2
$A_u^{(3)}$		213.8	212.0	199.9	207.6
$B_g^{(3)}$	221	214.8	212.0	199.9	207.6
$B_u^{(5)}$	221	216.9	212.0	207.3	215.1
$A_g^{(5)}$	228	223.8	224.8	213.0	224.1
$A_g^{(6)}$	249	242.5	244.4	231.8	241.6
$B_u^{(6)}$	252	246.1	244.4	231.8	241.6

Phonon band structure

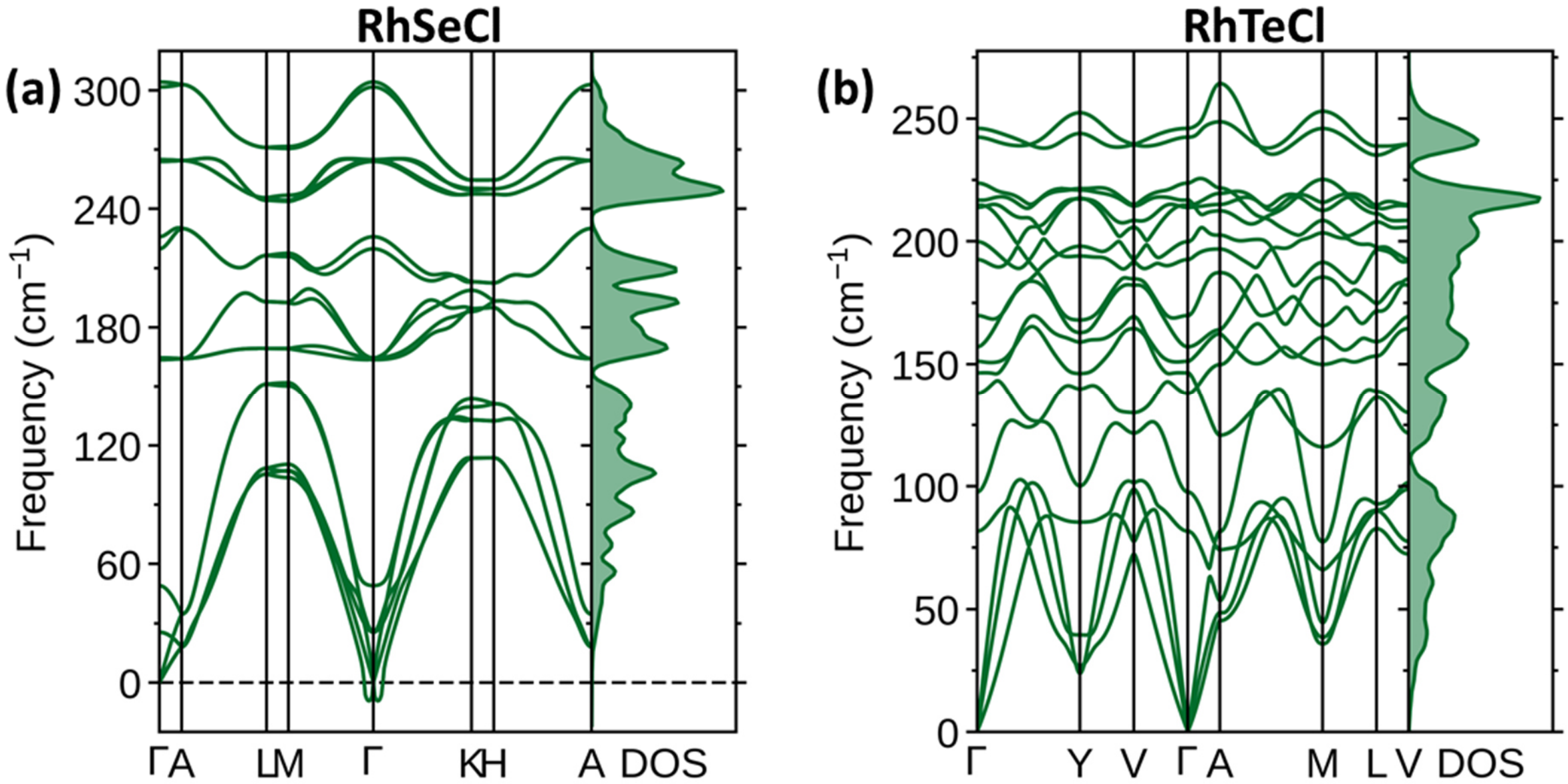

Figure S5. DFT-computed phonon band structure and vibrational density of states of RhSeCl and RhTeCl

Variable-temperature Raman measurements

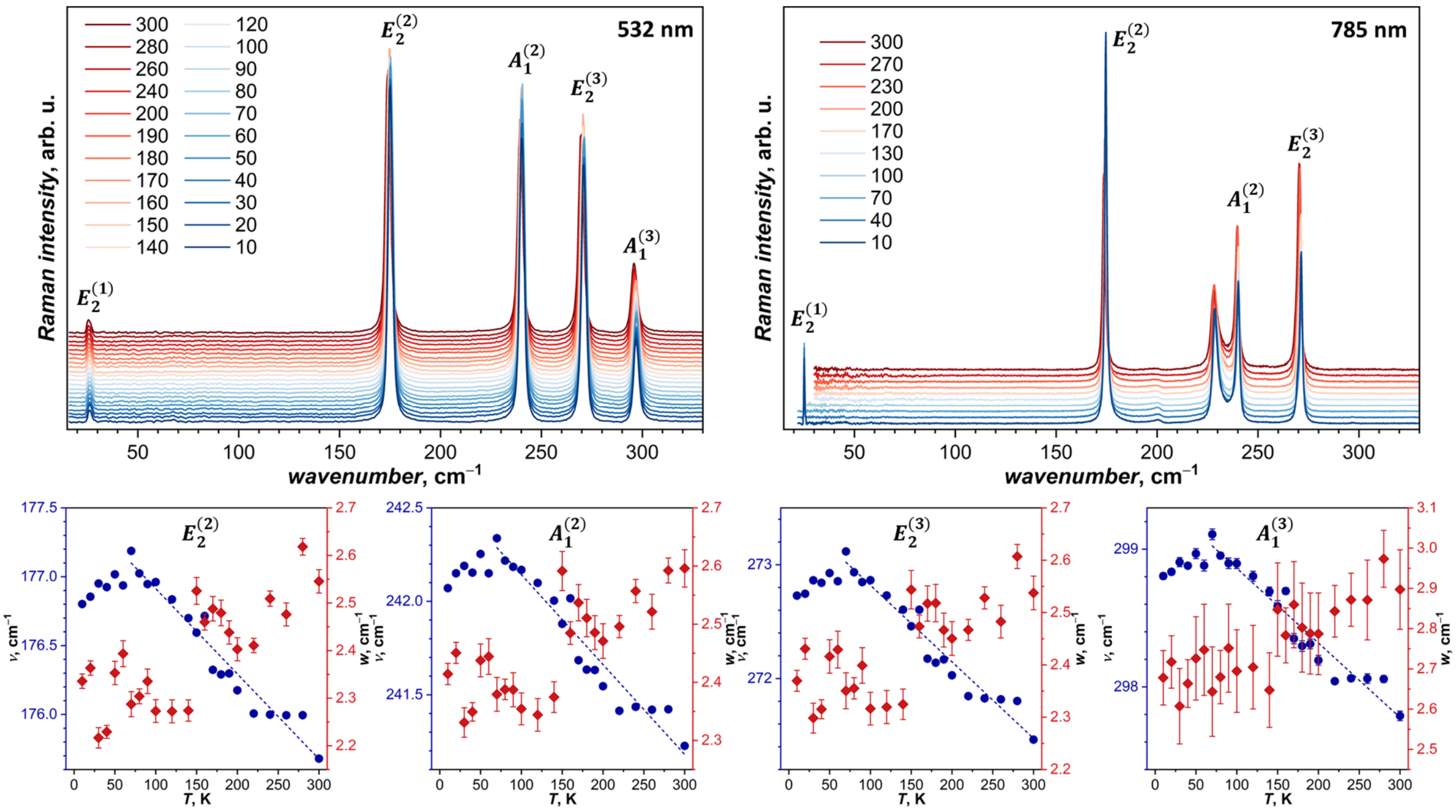

Figure S6. (Top) Variable-temperature Raman spectra of RhSeCl between 10 K and 300 K, excitation at 532 nm (left) and 785 nm (right). (Bottom) Temperature variation of the peak maximum (ν) and the half width at half maximum (w) for vibrational modes of RhSeCl.

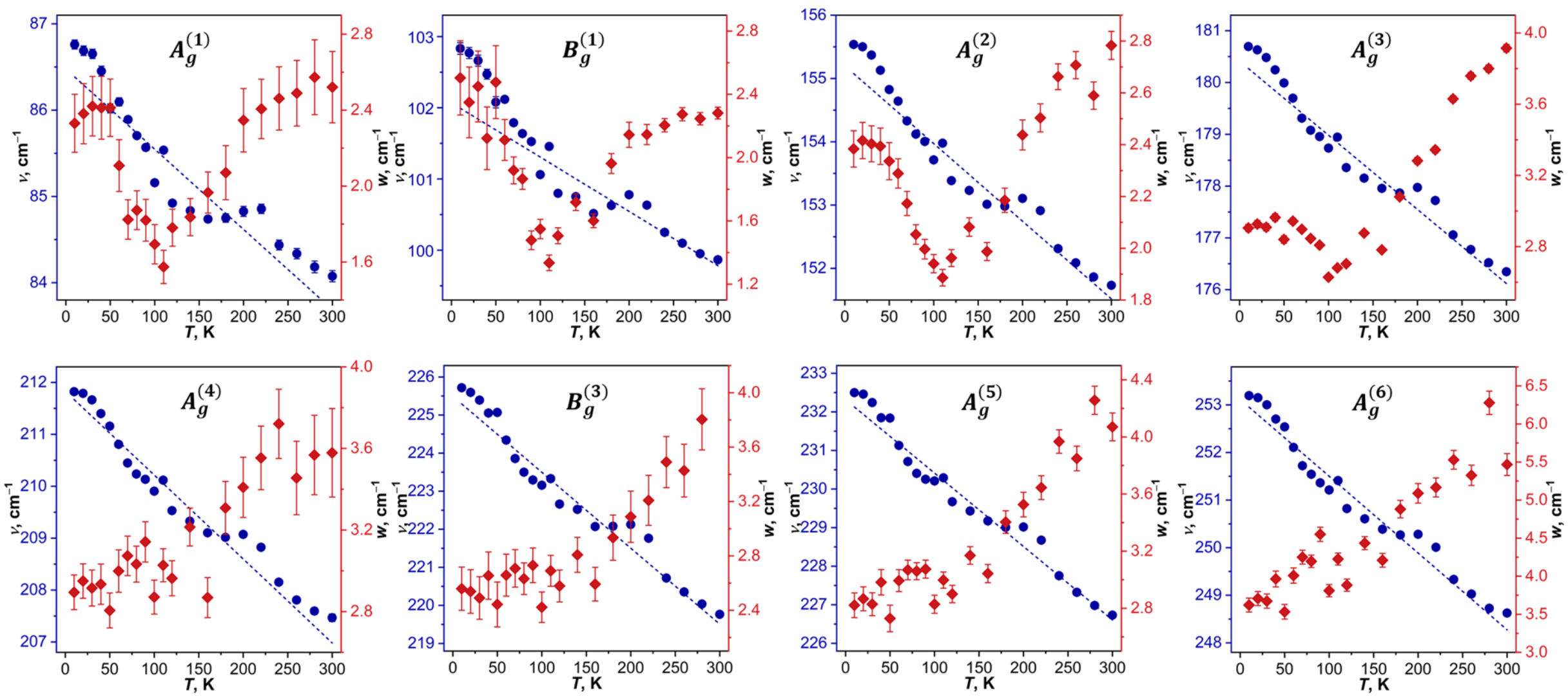

Figure S7. Temperature variation of the peak maximum (ν) and the half width at half maximum (w) for vibrational modes of RhTeCl.
